# Efficient Adsorption‐Based Direct Air Capture Via Triply Periodic Minimal Surface Architectures

**DOI:** 10.1002/advs.76487

**Published:** 2026-07-08

**Authors:** Qingyang Shao, Zhuozhen Gan, Chengcheng Long, Man Zhang, Yihe Miao, Yuehui Li, Xuancan Zhu

**Affiliations:** ^1^ Research Center of Solar Power & Refrigeration School of Mechanical Engineering Shanghai Jiao Tong University No. 800 Dongchuan Road Shanghai 200240 China; ^2^ College of Smart Energy Shanghai Jiao Tong University No. 800 Dongchuan Road Shanghai 200240 China

**Keywords:** adsorption, carbon capture and storage, chemical engineering, chemical separation, mass transfer

## Abstract

Direct air capture (DAC) of CO_2_ is a critical technology for climate change mitigation, yet its large‐scale deployment remains constrained by high energy demand and low process efficiency. In adsorbent‐based DAC systems, conventional contactor structures suffer from a fundamental trade‐off between adsorbent capacity and mass transfer kinetics, and the structure‐function relationship in CO_2_ capture has not been adequately elucidated. Here we demonstrate that this limitation can be overcome through the design of architected contactors enabled by the precision fabrication of topologies via additive manufacturing. Using 3D‐printed triply periodic minimal surface (TPMS) structures as active hydrodynamic substrates for DAC, we achieve a 70%–75% increase in the fraction of fast adsorption sites and a 114% enhancement in CO_2_ productivity compared with a conventional square‐channel monolith. The TPMS architecture induces vigorous chaotic advection and stable vortices that significantly thin the mass‐transfer boundary layer, alleviating the capacity–kinetics trade‐off, while simultaneously reducing energy consumption by 51.8%. These results suggest that architected flow topology can serve as a transferable design principle for improving DAC contactor efficiency.

## Graphical abstract

Architected triply periodic minimal surface (TPMS) contactors reshape direct air capture by replacing straight‐channel diffusion limits with convection‐enhanced CO2 transport. Chaotic advection thins boundary layers and exposes fast adsorption sites, enabling 70%–75% more fast sites, 114% higher productivity, and 51.8% lower energy consumption than conventional square channels for adsorbent‐based DAC operation.

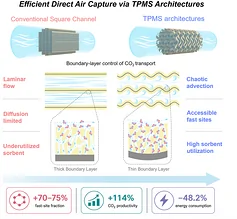

## 1 Introduction

Achieving net‐zero emissions necessitates not only the rapid decarbonization of energy systems but also the gigaton‐scale deployment of carbon dioxide removal technologies to address historical and residual emissions [1, 2, 3]. Among these, adsorption‐based direct air capture (DAC) stands out for its potential to utilize low‐grade heat and modular scalability [4, 5, 6, 7]. However, the industrial viability of DAC is critically constrained by the contactor inefficiencies arising from the gas‐solid interface between atmospheric CO_2_ in dilute 400 ppm concentrations and the adsorbent material [8, 9, 10, 11, 12, 13]. While significant advances have been made in synthesizing high capacity amine functionalized adsorbents, the translation of these material properties into system level performance remains inefficient due to the limitations of conventional contactors, making efficient contactor design critical to rendering the adsorption‐based DAC process less energy‐intensive and economically viable [14, 15, 16, 17, 18, 19].

The design of DAC contactors is governed by a persistent engineering dilemma [20, 21, 22]. While packed beds offer excellent mass transfer, they suffer from prohibitive pressure drops that drive up operational costs. Straight‐channel monoliths reduce hydraulic resistance by providing parallel flow paths, but their laminar flow fields can lead to thick concentration boundary layers and underutilization of adsorbent coatings [22, 23, 24, 25, 26, 27]. Fiber‐based sorbents represent another important contactor class, in which adsorbent‐containing polymeric or composite fibers can reduce inactive thermal mass and thereby lower sensible heat penalties [28, 29, 30]. However, these straight‐channel and fiber architectures rely primarily on axial flow with limited lateral mixing. Their CO_2_ transfer performance can remain constrained by external boundary layers and intracoating or intrafiber diffusion [22, 25, 31]. Especially when CO_2_ concentration falls from typical flue‐gas levels to ambient, the effective mass transfer coefficient can decline by nearly two orders of magnitude [25]. This diffusion limitation creates a severe bottleneck when scaling up capture capacity. To increase the volumetric capacity of a system, a common strategy is to thicken the adsorbent coating layer. However, in diffusion‐limited regimes, this additional capacity becomes effectively inaccessible. Due to the high internal diffusion resistance in thick coatings, accessing the deep adsorption sites requires prohibitively long contact times [29, 32]. Consequently, operators either terminate the cycle early to maintain speed, leaving a large portion of the adsorbent unutilized, or extend the cycle to saturate the bed, which drastically reduces the daily cycle count and overall productivity [25, 33, 34, 35, 36]. Thus, in current designs, high theoretical capacity rarely translates into high system performance [34, 37, 38].

Overcoming this limitation requires a paradigm shift from passive channel geometries to architected structures that actively modulate fluid dynamics to enhance mass transfer without incurring excessive pressure drops. While extensive research has focused on enhancing the intrinsic kinetics of adsorbents [7, 18, 39], the critical role of contactor hydrodynamics in realizing this potential remains underexplored. Additive manufacturing via 3D printing emerges as a transformative fabrication approach, enabling the creation of complex internal geometries that are unattainable via conventional methods [40, 41, 42, 43, 44, 45, 46, 47, 48]. Previous attempts, such as 3D‐printed helical channel contactors, have shown that inducing secondary Dean flow creates vortices that augment the CO_2_ transfer rate [34]. Bio‐inspired triply periodic minimal surfaces (TPMS) are recognized as a structural class with exceptional potential, characterized by a high surface area‐to‐volume ratio with mathematically continuous curvature and superior interconnectivity [49, 50, 51, 52]. In addition to their use as structured packings for solvent‐based CO_2_ absorption and in other adsorption‐related applications [50], TPMS architectures have recently been translated to solid‐sorbent DAC contactors. Polymer–sorbent DAC contactors were reported with complex TPMS geometries fabricated via templated phase inversion, demonstrating that PEI/silica TPMS contactors can deliver CO_2_ capture performance comparable to or better than other contactor geometries [53]. However, the structure–function relationship that links TPMS‐induced flow topology to adsorption kinetics under ultra‐dilute DAC conditions remains insufficiently resolved. In particular, how TPMS geometries modulate hydrodynamics to overcome mass‐transfer resistances in both the fluid boundary layer and the adsorbent interior has not been quantitatively elucidated. It is hypothesized that chaotic advection can enhance mass transfer rates significantly by transitioning the transport regime from diffusion‐dominated to convection‐enhanced. Theoretically, vigorous secondary flows and vortices actively disrupt the boundary layer, thereby facilitating convective transport to bulk adsorption sites. This hydrodynamic augmentation could enable the full utilization of high adsorbent loadings within abbreviated cycle times, alleviating the inherent trade‐off between capacity and adsorption kinetics.

Here, we provide a quantitatively grounded assessment of three distinct TPMS architectures for adsorption‐based DAC applications. First, a performance baseline was established using square‐channel monoliths to characterize the intrinsic trade‐off between capacity and kinetics. We then demonstrate how TPMS contactors, with comparable adsorbent loading, overcome this limitation. Compared to the square benchmark, TPMS structures delayed breakthrough time by 49%–54% and enhanced peak desorption concentration by 2.4–3.3 times. Further analysis revealed that TPMS contactors increase the fraction of fast adsorption sites by 70%–75%. These results clarify the mass transfer phenomena and uncover the mechanistic origins of TPMS superiority. Our findings demonstrate that 3D‐printed TPMS contactors act not merely as passive scaffolds, but as active hydrodynamic modulators that significantly enhance the mass transfer and thermodynamic efficiency of adsorption‐based DAC contactors.

## 2 Results and Discussion

### 2.1 Contactor Fabrication and Coating Performance

Four types of metallic substrates with square channels and three kinds of TPMS structures, Gyroid, Diamond, and Primitive, were fabricated using metal additive manufacturing (Figure 1a). Wall thickness (0.15 mm), relative density (9.75%), specific surface area (1.27 mm^2^/mm^3^), and hydraulic diameter (3 mm) of the four substrates were maintained at identical values. To evaluate the impact of adsorbent loading, square metallic substrates were coated with varying thicknesses of 3, 6, and 9 dip‐coating cycles, designated as Square−, Square, and Square+, respectively (Figure S6). Additionally, three distinct TPMS substrates underwent 6 dip‐coating cycles to achieve adsorbent loadings comparable to the standard Square contactor (Figure S7). All coating processes were conducted in triplicate.

**FIGURE 1:**
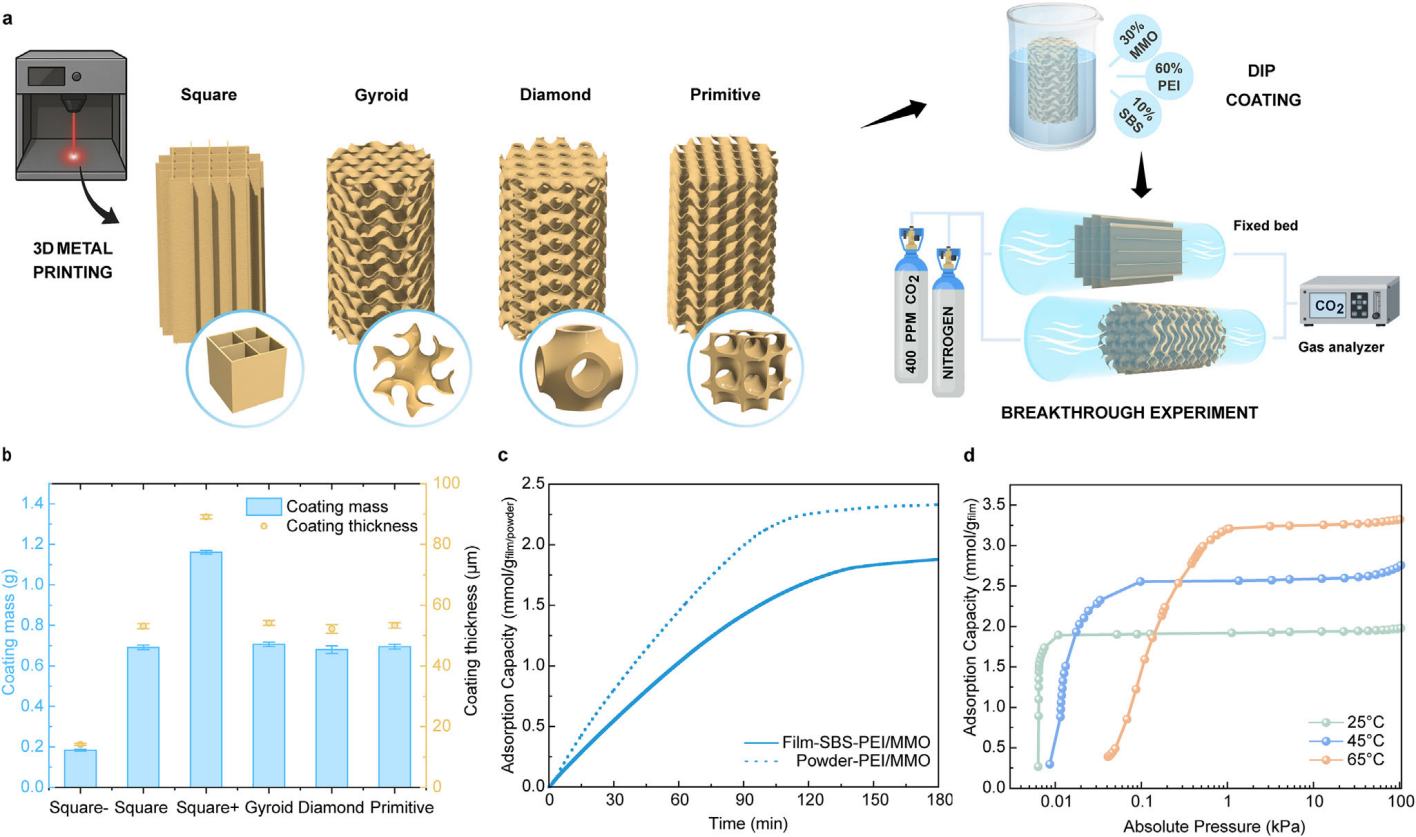
3D printing contactor and coating adsorbent. a, Scheme of contactor fabrication procedure. b, Adsorbent coating mass and reference thickness of contactors. c, Dynamic adsorption curves of adsorbent powder (dotted line) and coating (added with 10 wt.% SBS binder, solid line). d, CO_2_ adsorption isotherms of adsorbent film (25°C, 45°C, and 65°C).

Notably, the mass deviation between the TPMS structures and the Square contactor remained below 3%. This strict mass control validates the subsequent comparison of adsorption performance across different contactors. Given the intricate topology of contactors, which precludes accurate direct measurement, the coating thickness was derived indirectly. The coating density was experimentally determined using detached coating samples. The reference thickness was calculated (Equation S1) based on the surface area of the substrate and the coating mass, as shown in Figure 1b. While it is acknowledged that the complex topology of TPMS may induce local variations in coating thickness (e.g., accumulation in nodes), the rigorous control of total adsorbent mass (<3% deviation) and identical geometric surface areas ensures that the observed performance differences can be primarily attributable to the hydrodynamic influence of the contactor architecture rather than material loading discrepancies. The resulting reference thicknesses for the Square series were 14.1, 53.1 and 89.1 µm, whereas the TPMS structures exhibited thicknesses of 54.2, 52.2 and 53.3 µm.

The SEM image of the Film‐SBS‐PEI/MMO coating adsorbent (Figure S1) shows that the microstructure of the coating is consistent with that of the adsorbent powder (Figure S2). In the specific surface area and pore size analyses of the Film‐SBS‐PEI/MMO coating adsorbent (Figure S3 and Table S1), the specific surface area of the adsorbent powder slightly decreased after the addition of the 10 wt.% SBS binder, while the pore volume remained the same. According to the pore size distribution, the pores in the coating were predominantly below 5 nm. The addition of the binder did not significantly alter the morphology of the adsorbent, indicating that the SBS was well‐dispersed between the particles and did not cause severe blockage of the pore structure.

The CO_2_ adsorption performance of the adsorbent powder and the coating layer were tested. The adsorption curves obtained using TGA (Figure 1c) showed that the CO_2_ uptake of the Film‐SBS‐PEI/MMO was 1.88 mmol/g within 180 min, representing a retention of 80.3% of the capacity of the Powder‐PEI/MMO. The CO_2_ adsorption isotherms of Film‐SBS‐PEI/MMO were measured at 25, 45, and 65 °C over a pressure range of 0–100 kPa using the static volumetric method (Figure 1d). At 25°C and a CO_2_ partial pressure of 0.4 kPa, the CO_2_ capacity of 1.90 mmol/g is consistent with that from the thermogravimetric method. While the addition of SBS as a binder dilutes the active components, the performance decrease is considered acceptable.

### 2.2 Dynamic Breakthrough Experiment

CO_2_ breakthrough experiments were conducted in a custom fixed‐bed reactor using cylindrical contactors of 18 mm diameter and 30 mm height. A 400 ppm CO_2_/N_2_ mixture was introduced at 1 or 2 L/min over a 3‐hour adsorption period, corresponding to superficial velocities of 0.066 and 0.131 m/s, respectively, based on the external cross‐sectional area of the contactor. Coating uniformity for each structure is provided in Table S2.

Breakthrough behavior was characterized by the time points at which outlet CO_2_ reached 5% (*t*_5,_ breakthrough point), 50% (*t*_50_, half‐saturation point), and 95% (*t*_95,_ saturation point) of the inlet concentration. It should be noted that *t*_5_ is not intended to define the practical termination point of a DAC adsorption cycle, since the outlet gas below the inlet CO_2_ concentration still represents partial CO_2_ capture. Instead, *t*_5_ is used here as a kinetic descriptor of the initial high capture efficiency state. At *t*_5_, the instantaneous CO_2_ removal efficiency remains approximately 95%, and this point therefore marks the onset of CO_2_ leakage and the leading edge of the mass transfer zone. As shown in Figure 2a, both *t*_5_ and *t*_95_ followed the trend of Square+ > Square > Square−. At 1 L/min, increasing the adsorbent loading from Square− to Square by 3.7‐fold extended *t*_5_ from 2.6 to 17.6 min, a 6.7‐fold improvement. However, a further 1.68‐fold loading increase for Square+ only raised *t*_5_ to 20.4 min, indicating diminishing returns likely due to internal diffusion resistance. Higher loadings also prolonged saturation time (*t*_95_), leading to more pronounced tailing in breakthrough curves. Although tailing enhances total CO_2_ capture, the low capture rate in this stage may reduce cyclic system efficiency. At 2 L/min, *t*_5_ values decreased to 1.1, 2.2, and 2.6 min, respectively, while tailing diminished significantly.

**FIGURE 2:**
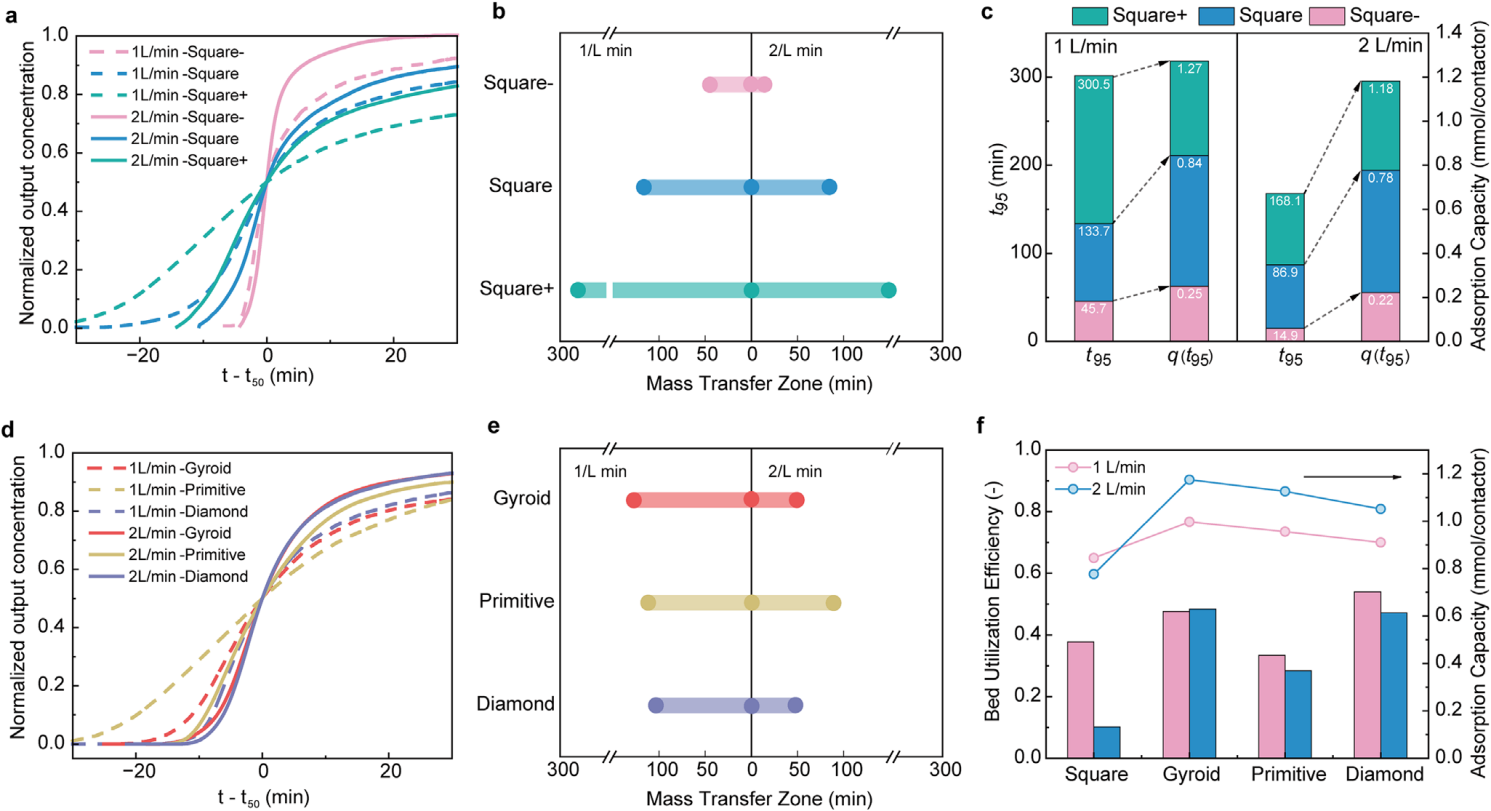
Dynamic breakthrough performance. a, Breakthrough curves for square‐channel contactor under 1 and 2 L/min. b, The MTZ for square‐channel contactor. c, Paired relationship between saturation time (t95) and CO_2_ uptake obtained at the corresponding saturation time, q(t95), for square‐channel contactors. d, Breakthrough curves of contactors with different structures under 1 and 2 L/min. e, The MTZ width for different structures. f, Adsorption capacity per unit volume and bed utilization efficiency of contactors with different structures.

The mass transfer zone (MTZ) length, defined as *t*_95_ − *t*_5_, reflects the adsorption kinetics. As plotted in Figure 2b, the MTZ width increased in the order of Square+ > Square > Square−. At 1 L/min, the 3.7‐fold loading increase from Square− to Square widened the MTZ from 43.1 to 116.1 min. In contrast, Square+ exhibited a 5.3‐fold higher loading and an over 5.5‐fold wider MTZ than Square−, indicating that thicker coatings significantly extend the transition zone between saturated and fresh adsorbent. This broadening MTZ reflects slower adsorption front propagation and increased diffusion resistance. Thicker coatings elongate the diffusion path and raise tortuosity, thereby reducing mass transfer rates and bed utilization efficiency. Ideally, negligible mass transfer resistance would yield an MTZ approaching zero, with a sharp breakthrough front.

The volumetric CO_2_ uptake integrated from breakthrough curves to *t*_95_ reveals a distinct capacity and kinetics trade‐off across structures with different coating loadings (Figure 2c). As loading increased from Square− to Square+, the adsorption capacity rose from 0.25 mmol to 1.27 mmol, while *t*_95_ extended from 45.7 to 300.5 min. This trend indicates that increasing coating thickness improves total capacity but also substantially slows the approach to saturation, owing to increased diffusion resistance within the coating layer. Within the tested square‐channel series, the intermediate‐loading Square contactor exhibited the highest average adsorption rate under the present experimental conditions, as calculated from the integrated CO_2_ uptake divided by the corresponding saturation time. This observation highlights the limitation of the conventional loading‐driven strategy. Although thickening the adsorbent layer in straight channels (Square+) does raise the total and volumetric capacity, much of the added coating remains kinetically inaccessible within practical cycle times, because the deep coating is shielded by the laminar boundary‐layer and intracoating diffusion resistance. As a result, the added capacity is not efficiently converted into faster, more energy‐efficient capture without a concurrent enhancement in mass transfer. A true optimization would require an economic analysis beyond the scope of this study.

Breakthrough curves of TPMS‐structured contactors were compared with a square‐channel design at equivalent adsorbent loadings (Figure 2d). Gyroid and Diamond geometries exhibited delayed breakthrough relative to the square channel, indicating higher effective capacity. At 1 L/min, *t*_5_ values for Gyroid and Diamond were 26.2 and 27.1 min, respectively, 49% and 54% longer than that of Square. The complete characteristic breakthrough times (*t*_5_, *t*_50_, and *t*_95_) for all TPMS and Square contactors at both flow rates are summarized in Table S3. This enhancement likely stems from the tortuous flow paths in Gyroid and Diamond, which promote eddy formation, redirect axial momentum into tangential motion, and intensify mass transfer within the adsorbent layer, thereby improving access to CO_2_ adsorption sites. In contrast, Primitive showed a breakthrough time similar to Square, likely due to its higher permeability and curved surface connections that hinder vortex generation. Nonetheless, its capture efficiency still surpassed that of Square. Saturation times were comparable across all structures.

Interestingly, the variation of flow rate has different effects on contactors of different structures. Since increasing the volumetric flow rate is expected to shorten the breakthrough time, the more informative comparison is the flow‐rate dependence of the integrated adsorption capacity and bed utilization. For the Square contactor, increasing the flow rate from 1 to 2 L/min reduced the adsorption capacity by 8%, suggesting that the shorter residence time aggravates external mass‐transfer limitations in the straight‐channel geometry. In this case, axial convection becomes dominant, whereas radial diffusion across the boundary layer toward the adsorbent coating is insufficient within the available contact time. By contrast, all TPMS contactors exhibited an approximately 17% increase in adsorption capacity at 2 L/min, indicating that the superior ability of TPMS structures to resist rapid breakthrough and maintain high capture efficiency under high flow. The continuously curved and interconnected TPMS geometries promote secondary flow, and boundary‐layer disruption, enabling more efficient utilization of the adsorbent layer even under shortened residence time.

Notably, the more pronounced tailing of the Primitive contactor at 2 L/min may arise from its relatively high axial continuity and limited lateral mixing. Compared with Gyroid and Diamond, the Primitive topology contains more direct through‐passages, while transverse communication mainly occurs through side‐connected curved openings. At high flow rate, the shortened residence time reduces the opportunity for gas to exchange laterally through these side openings. Consequently, part of the gas preferentially follows the more open axial passages, whereas laterally connected adsorbent regions are accessed more slowly. This broadens the MTZ and produces a less uniform adsorption front, leading to a tailing.

Furthermore, the Gyroid and Diamond contactor exhibits the narrowest MTZ (Figure 2e), demonstrating superior mass transfer kinetic performance and high utilization of the adsorbent bed. In contrast, the simple straight channels of Square structure facilitate the formation of stable laminar flow, leading to a thicker mass transfer boundary layer and greater external diffusion resistance. Overall, TPMS architectures generally possess advantages in enhancing mass transfer, an advantage that becomes more pronounced at higher flow rates.

At 1 L/min, the CO_2_ adsorption capacities of the Gyroid, Primitive and Diamond structured contactors were 0.997, 0.956, and 0.910 mmol respectively, representing increases of 18%, 13% and 7.7% compared to the Square structured contactor (Figure 2f). This enhancement stems from the intrinsic tortuosity of TPMS channels, which disrupts laminar flow and induces vortices. This complex flow pattern enhances convective transport, renews the gas near the adsorbent surface, and reduces concentration boundary‐layer resistance. The structural advantage of TPMS became more pronounced at 2 L/min, where all TPMS contactors exhibited a further 17% increase in capacity, contrasting with an 8% decrease for the Square structure.

### 2.3 Enhancement Mechanism of TPMS Geometries

To understand the fundamental advantage of TPMS, we must first analyze the governing transport phenomena. In DAC, the capture process involves three steps: external diffusion across the boundary layer, internal pore diffusion, and surface chemical reaction. Given the ultra‐low partial pressure of CO_2_, the external diffusion is often the rate‐limiting step [25]. Therefore, the optimal contactor design must minimize the boundary layer thickness while maximizing the interfacial contact area. Unlike straight channels where gas streamlines run parallel to the wall limiting mass transfer to slow lateral diffusion, the continuously curving walls of TPMS structures force the bulk gas flow to impinge directly upon the adsorbent surface. This advective transport effectively bypasses the slow diffusion process, theoretically allowing for a transition from a diffusion‐dominated regime to a convection‐enhanced regime. We hypothesize that this mechanism is the primary driver for utilizing the bulk adsorption sites that are typically inaccessible in conventional designs.

The CFD simulation results provide insight into the hydrodynamic behavior within the contactor, as illustrated by the streamlines in Figure 3a to Figure 3c. The fluid exhibits a highly tortuous flow path dictated by the interconnected pore topology of the TPMS structure. Unlike simple straight channels, the complex geometry forces the fluid to undergo continuous splitting and recombination, thereby promoting significant lateral mixing and reducing the boundary layer thickness. The uniform distribution of streamlines suggests that the TPMS architecture effectively minimizes flow channeling and dead zones. Quantitative analysis of the flow field, specifically the vorticity magnitude presented in Figure S16, reveals distinct hydrodynamic differences between the architectures. The TPMS contactors exhibit significantly elevated vorticity levels compared to Square contactor, which induce stronger rotational fluid motion and shear stress as the gas traverses the channels. Notably, the Gyroid structure outperforms the other evaluated geometries, yielding the highest average vorticity. Such intense vorticity implies the generation of robust local mixing, which is critical for disrupting the boundary layer and enhancing interfacial mass transfer efficiency. Because the Square and TPMS substrates share the same relative density and external dimensions (Table 1), their open volume and mean gas residence time are essentially identical. The pronounced differences in CO_2_ breakthrough therefore stem from transverse, wall‐directed transport. As indicated by the vorticity fields (Figure S16), this process continuously thins and renews the concentration boundary layer, rather than arising from variations in bulk gas residence time.

**FIGURE 3:**
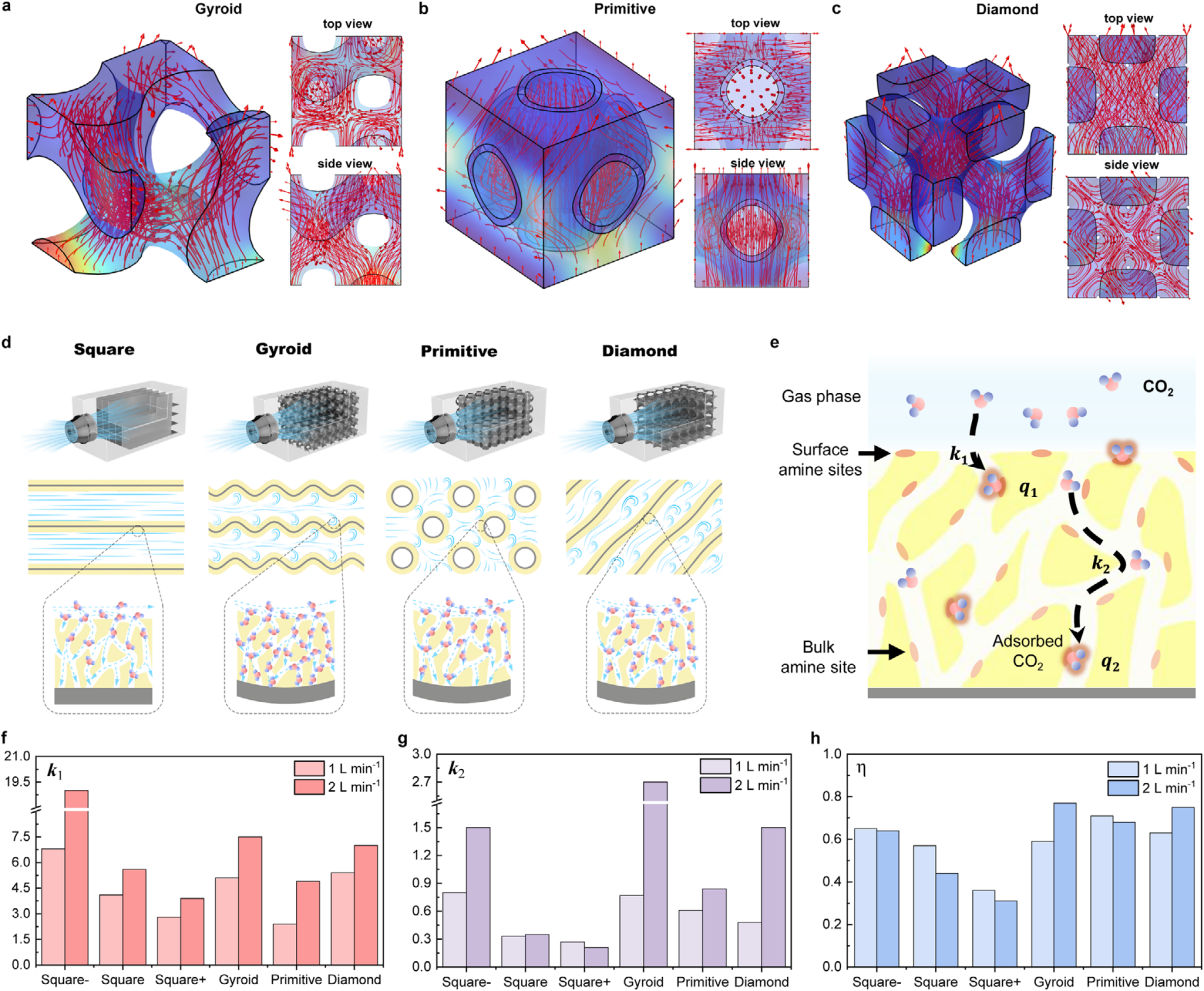
Enhancement mechanism of TPMS geometries. a–c, Visualization of streamlines within the TPMS contactor obtained from CFD simulations. d, Mechanistic illustration of flow and mass transfer pattern of each contactor. e, Mechanism of the dual kinetic model. f–h, The fitted mass transfer parameter values of *k*_1_, *k*_2_, *η*.

**TABLE 1:** Geometric parameters of Square and TPMS substrate.

Substrate	Pore diameter/Cell size (mm)	wall thickness (mm)	relative density (%)	specific surface area (mm^2^/mm^3^)	hydraulic diameter (mm)
Square	3	0.15	9.75	1.27	3.15
Gyroid	4.85	0.15	9.75	1.27	3.15
Primitive	3.7	0.15	9.75	1.27	3.15
Diamond	6.05	0.15	9.75	1.27	3.15

The CFD simulations provide the physical basis for the model results. To quantify the effects of adsorbent coating thicknesses and contactor structure on adsorption performance, the DK model (Equation (4) and Equation (5)) was established to fit the breakthrough curves of each contactor at various flow rates. As illustrated in Figure 3e, the DK model conceptualizes adsorption at two distinct sites: Fast, easily accessible surface sites and slow, diffusion‐limited bulk sites within the interior of the adsorbent. The model accurately captures the tailing phenomenon by attributing it to slow kinetics at these bulk sites [25, 33, 34]. The model‐fitted breakthrough curves (Figures S12 and S13) and corresponding *R* ^2^ values (Table S9) show a high degree of consistency between the model calculations of the experimental data, indicating the model accurately reflects contactor adsorption behavior under different conditions. The fitted mass transfer parameters derived from the DK model for each contactor under various flow rates are presented in Figure 3f to h and Table S10.

A comparison of contactors with varying adsorbent loadings reveals that *η* decreases with increasing coating thickness. Moreover, both *k*_1_ and *k*_2_ decrease as the adsorbent coating thickness increases. This finding underscores a dual limitation imposed by coating thickness. It not only alters the proportion of fast versus slow adsorption sites but also impedes the intrinsic adsorption kinetics at these sites due to increased intraparticle diffusion resistance.

An increase in the gas flow rate results in higher values for both *k*_1_ and *k*_2_ across all contactors. This is attributed to enhanced external mass transfer, where a higher volumetric flow supplies more gas molecules to the adsorbent surface per unit of time. However, for the square‐channel contactors, an increase in flow rate leads to a further reduction in *η*. This phenomenon occurs because higher flow rates reduce the residence time of CO_2_ within the channels. Under these conditions, axial convection dominates over radial diffusion toward the adsorbent layer. Gas molecules possess insufficient time for deep penetration into the coating, thereby reducing the number of accessible fast adsorption sites.

In contrast, TPMS‐structured contactors exhibit high *η* values of 0.7 at the same adsorbent loading. Opposing the trend in straight channels, the *η* value for TPMS structures increases to 0.8 at higher flow rates. Structural comparisons reveal that the *k*_1_ and *k*_2_ values for the Gyroid and Diamond contactors are consistently higher than those for the Square contactor. Both *k*_1_ and *k*_2_ are apparent lumped coefficients in which the external gas‐film resistance acts in series with the respective internal resistance. CO_2_ destined for the bulk amine sites must first cross the same external boundary layer before diffusing into the coating. The TPMS‐induced thinning and continuous renewal of this boundary layer therefore raises both coefficients, without altering the intrinsic diffusivity of the identical coating. The fast‐site pathway (*k*_1_) is dominated by rapid chemisorption on readily accessible surface sites and already operates close to its kinetic ceiling in all geometries, so it gains comparatively little; the slow‐site pathway (*k*_2_) is bottlenecked by the sustained supply of CO_2_ to and into the coating interior and therefore has far more headroom. The larger relative enhancement of *k*_2_ is thus the expected signature of an external‐transport enhancement acting on two pathways with different rate‐limiting steps. This demonstrates that TPMS geometries enhance flow patterns and mass transfer characteristics, playing a crucial role in governing the overall adsorption kinetics.

### 2.4 Desorption Via Temperature Swing

In the desorption process, the bed was heated to 120°C under a 100 mL/min N_2_ purge for 1 h. As adsorbent loading increased, the peak CO_2_ desorption concentrations for the Square−, Square, and Square+ straight‐channel contactors were 3900, 8600, and 8700 ppm, respectively (Figure 4a). The Square− and Square contactors exhibited similar initial desorption rates, whereas that of Square+ was significantly lower. Interestingly, the peak CO_2_ desorption concentrations for the Gyroid, Primitive, and Diamond contactors were 28,700, 20,800, and 23,500 ppm, respectively (Figure 4b), which are 3.3, 2.4, and 2.7 times higher than the Square contactor.

**FIGURE 4:**
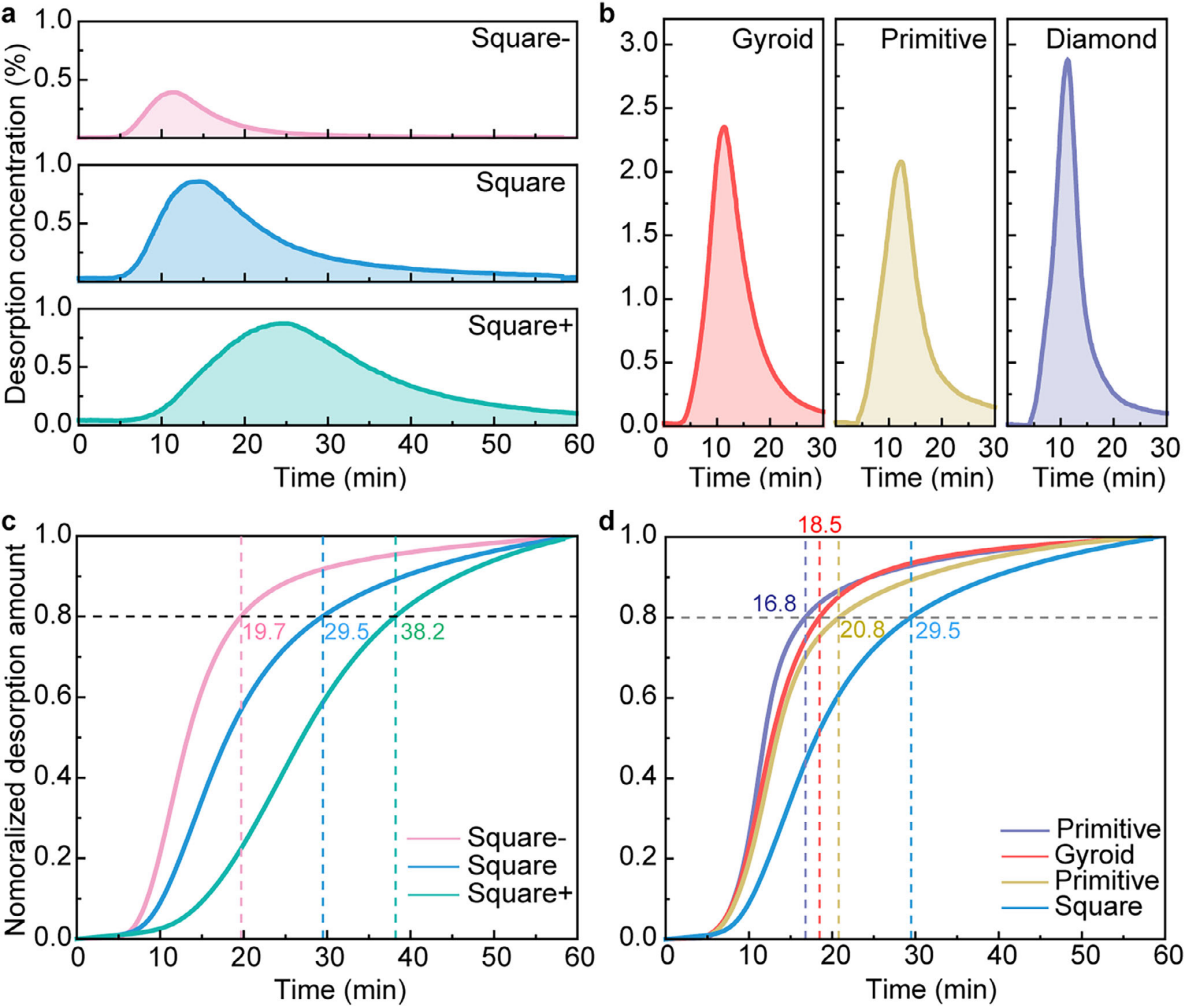
Desorption experiment. a, Desorption curves of square‐channel contactors. b, desorption curves of TPMS contactors. c, Normalized desorption capacity of square‐channel contactor. d, Normalized desorption capacity of TPMS contactor.

By integrating the desorption curves, the amount of desorbed CO_2_ was plotted against time and normalized to identify the relative desorption rates of the contactors (Figure 4c). The time required for the Square−, Square, and Square+ contactors to reach 80% of total desorption was 19.7, 29.5, and 39.2 min, respectively. The longer desorption time of Square+ reflects its thicker coating, which lengthens the intracoating diffusion path, thereby slowing the desorption rate. Notably, the times required for the Gyroid, Primitive, and Diamond contactors to reach 80% total desorption were 16.8, 20.8, and 18.5 min, respectively (Figure 4d), representing improvements of 43%, 30%, and 37% over the Square contactor. It is worth noting that desorption time does not indicate overall performance, because productivity is governed by the amount of CO_2_ captured per cycle relative to the total cycle time. This point will be discussed in detail in the subsequent Performance evaluation section.

The faster desorption of the TPMS contactors should be interpreted as an apparent regeneration enhancement arising from mass‐transfer effects. In the present experiment, the reactor wall was maintained at 120 °C by a circulating oil bath, while the N_2_ purge gas was preheated to the same temperature. Heat was therefore delivered both by conduction from the fixed‐bed wall to the contactor and by convection from the heated purge gas. Under identical external heating and purge conditions, the present experiments were not designed to independently measure local temperature fields or extract structure‐specific heat‐transfer coefficients, but two simple estimates separate their roles here. First, the contactors are small and highly conductive (AlSi10Mg, k ≈ 150 W/(m·K), ∼150 µm walls). The characteristic conduction time (≈L^2^ρcp/k) is only ∼0.4 ms through the wall and ∼1–2 s across the 9 mm radius. This is far shorter than the tens‐of‐minutes desorption. So each contactor reaches the 120 °C set temperature almost instantly, and identically for all geometries (same material and wall thickness). Second, the N_2_ purge is preheated to the regeneration temperature and therefore delivers negligible heat (an upper bound of ∼0.2 W even if it entered cold), serving only to sweep desorbed CO_2_ away (Note S4). These estimates indicate that geometry‐dependent heat‐transfer differences are unlikely to dominate the observed desorption‐rate differences in the present small metallic contactors. The faster apparent regeneration of TPMS contactors is therefore primarily attributed to enhanced mass‐transfer and sweep‐gas distribution under flowing N_2_ regeneration. For larger contactors or steam regeneration, where heat transfer becomes significant and geometry‐dependent, a dedicated heat‐ and mass‐transfer decoupling study will be required. For practical steam‐assisted TSA or steam‐assisted TVSA operation, the same hydrodynamic principle is expected to remain relevant because steam also functions as a flowing sweep medium while simultaneously supplying heat for regeneration. Therefore, the TPMS‐induced enhancement in gas‐phase renewal and boundary‐layer disruption should also assist CO_2_ removal under steam purge conditions. Nevertheless, the behavior of TPMS contactors under practical steam‐purge regeneration conditions requires further investigation in future work. For operation without an external sweep gas, particularly in larger or less thermally conductive contactors, where heat transfer rather than gas–solid contacting becomes rate‐limiting, the expected benefit of TPMS geometries would arise mainly from improved heat transfer during the heating step [54, 55, 56, 57], rather than from convective sweep‐gas mixing. Accordingly, the present desorption results should be interpreted as evidence that TPMS architectures can accelerate desorption under flowing regeneration conditions.

### 2.5 Performance Evaluation of Scalable TPMS Contactors

To further assess the implications of the experimentally observed mass‐transfer enhancement under more industrially relevant gas throughput, a scaled‐up contactor model with a length of 300 mm was utilized for performance evaluation. The superficial velocity in this scaled‐up model was set to 1.3 m/s, which is within the range relevant to practical DAC operation. This model‐based analysis combines the experimentally fitted adsorption kinetics with pressure‐drop and fan‐energy contributions as a scale‐up performance assessment. CO_2_ productivity is a key metric for evaluating contactor performance. It is defined as the amount of CO_2_ captured (working capacity, *q*_ads_) per unit volume of the contactor (*V*_contactor_) per unit of total cycle time (*t*_cycle_ = *t*_ads_ + *t*_des_). The corresponding mass‐normalized productivities, which account for the total contactor mass governing the sensible‐heat penalty, are summarized in Table S17. A systematic analysis of different interface conditions is performed. Figure 5a shows variations in CO_2_ productivity with fractional adsorption time (defined as the ratio of the adsorption time to the total cycle time, *t*_ads_/*t*_cycle_), while holding fractional desorption time fixed at *t*_90_ (the time required to achieve 90% of the total desorption). A consistent trend emerges that productivity decreases as adsorption time increases from *t*_50_ across all contactors, primarily attributed to adsorption kinetics. For instance, in the Gyroid structure, increasing adsorption time from *t*_50_ to *t*_90_ decreased CO_2_ productivity from 145.8 to 91.1 kg/m^3^/d. A shorter adsorption time maximizes operation in the most efficient rate range, enabling more cycles and higher average daily productivity.

**FIGURE 5:**
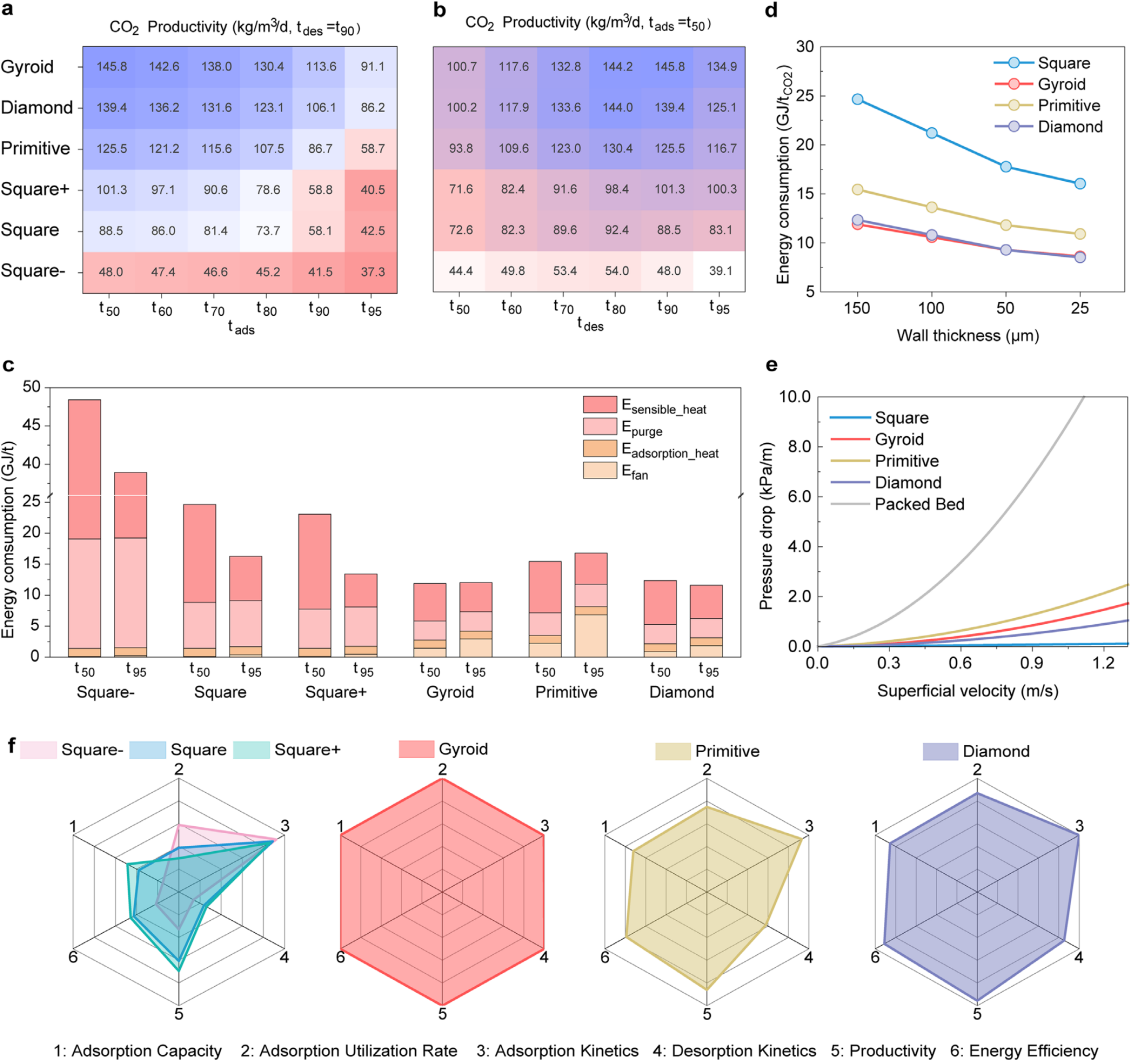
Contactor performance evaluation. a, CO_2_ productivity at different fractional adsorption loadings. b, CO_2_ productivity at different fractional desorption loadings. c, energy consumption, and its components (where *E*_fan_ is electrical energy consumption and the rest are thermal energy consumption). d, Sensitivity analysis of total energy consumption to wall thickness of metal substrate. e, Pressure drop as a function of superficial velocity for structured contactors and a representative packed bed. f, radar charts of key performance indicators.

Comparing contactor structures, CO_2_ productivity followed the order: *P*_Gyroid_ > *P*_Diamond_ > *P*_Primitive_ > *P*_Square+_ > *P*_Square_> *P*_Square−_. Notably, TPMS‐structured contactors exhibited markedly enhanced productivity compared to Square contactors, with *P*_Gyroid_ achieving a 65%–114% improvement over *P*_Square_. This highlights the superior mass transfer performance of TPMS structures, a core advantage for high productivity. Importantly, the TPMS contactors also showed substantial productivity improvements over Square+, even though Square+ contained approximately 68% more adsorbent than the standard Square and TPMS contactors.

The effect of desorption time on productivity was also investigated (Figure 5b), with adsorption time fixed at *t*_50_. Productivity of all contactors displayed an optimal desorption time range. For example, the productivity of Diamond structure peaked at 144.0 kg/m^3^/d near *t*_80_. This reflects a trade‐off between regeneration degree and cycle speed. An overly short *t*_des_ at *t*_50_ causes insufficient regeneration, reducing effective adsorption capacity in the subsequent cycle and limiting capture per cycle and overall productivity. Conversely, an overly long *t*_des_ at *t*_95_ results in excessive desorption. While regeneration is high, the marginal gain beyond *t*_80_ is minimal due to slow desorption rate at later times. This unnecessarily prolongs downtime, reducing daily cycle count and lowering average productivity. Overall, *t*_des_ in the range of t_70_–t_90_ represents the sweet spot, balancing sufficient regeneration for efficient adsorption with reasonable cycle frequency. Across desorption strategies, TPMS contactors maintained significant advantages over square‐channel designs. Specifically, the Gyroid achieved 145 kg/m^3^/d at its optimal desorption time, while the Square structure reached only 92.4 kg/m^3^/d.

Figure 5c illustrates the specific energy consumption profiles and their constituent breakdowns at two distinct fractional adsorption times: breakthrough time (*t*_ads_ = *t*_50_) and saturation time (*t*_ads_ = *t*_95_) under fixed desorption conditions (*t*_des_ = *t*_80_). The total specific energy consumption comprises four components: sensible heat for the adsorbent and contactor (*E*_sensible_heat_), heat required for the purge gas (*E*_purge_; *note: While steam purging is more common, nitrogen purging in this study incurs analogous heat consumption. This is a conservative estimate to facilitate a rigorous comparison*.), the heat of desorption for CO_2_ (*E*_adsorption_heat_), and fan energy (*E*_fan_). It should be noted that the values reported here correspond to a gross, no‐heat‐recovery energy demand under dry N_2_‐purge regeneration, providing an internally consistent comparison among contactor structures. No sensible heat recovery from the hot purge gas, the desorbed gas stream, or the heated contactor/adsorbent matrix was assumed. This unintegrated energy accounting leads to higher absolute energy values than process‐level analyses that include steam regeneration, heat integration, or purge‐gas heat recovery.

A distinct divergence in energy trends is observed between the contactor geometries. For the square‐channel contactors (Square−, Square, and Square+), extending the adsorption duration from *t*_50_ to *t*_95_ yields a consistent and substantial reduction in total specific energy. Specifically, the energy consumption for the standard Square contactor declines from approximately 24.7 at *t*_50_ to 16.3 GJ/t at *t*_95_. This reduction is primarily driven by the amortization of fixed thermal investments—namely *E*_sensible_heat_ and *E*_purge_—over a larger mass of captured CO_2_. Since these thermal penalties are relatively constant per cycle, maximizing the working capacity per cycle effectively dilutes the thermal energy cost per tonne of product. Conversely, *E*_sensible_heat_ remains nearly constant per cycle under fixed contactor mass and temperature swing.

In contrast, the TPMS‐structured contactors exhibit a different behavior. While they achieve significantly lower overall energy consumption than the square geometries, extending the adsorption time does not yield the same magnitude of reduction; in the case of the Primitive, the specific energy even increases at *t*_95_. This phenomenon is attributed to the trade‐off between thermal efficiency and hydraulic resistance. While TPMS structures minimize thermal mass, they induce complex flow patterns that increase pressure drop (the following text will provide a detailed discussion of Figure 5e). Consequently, at prolonged adsorption times (*t*_95_), the cumulative fan energy consumption (*E*_fan_) becomes non‐negligible, offsetting the benefits of thermal amortization. Despite the fan energy penalty, TPMS‐based contactors demonstrate superior energy efficiency overall. At *t*_50_, the Gyroid contactor consumes approximately 11.9 GJ/t, representing only 48.2% of the energy required by the Square contactor (24.7 GJ/t). This efficiency gain is fundamentally underpinned by the superior mass transfer characteristics of TPMS topologies relative to square channels. Within identical fractional adsorption times, the enhanced mass transfer facilitates the capture of a significantly larger absolute quantity of CO_2_. Consequently, the fixed sensible heat investment (*E*_sensible_heat_) is amortized over a greater mass of captured product, resulting in a markedly lower specific thermal load. In industrial settings utilizing steam purging, similar flow‐renewal benefits may be expected. The reduced dead‐zone tendency of TPMS structures may enable more uniform steam distribution and potentially more efficient steam utilization than square channels, potentially reducing the parasitic thermal load beyond the conservative estimates presented here. It should be emphasized that the present productivity and energy analysis was conducted under dry conditions. H_2_O co‐adsorption/desorption, steam condensation, and humid heat‐transfer effects were not included, which will be discussed in future studies.

Juxtaposing this energy consumption analysis with the productivity results reveals a critical optimization trade‐off. For the conventional Square contactor, high productivity (at *t*_50, ads_) comes at the cost of high energy consumption, or vice versa. In marked contrast, TPMS contactors mitigate this trade‐off by facilitating rapid adsorbent utilization. This capability allows TPMS structures to maintain significantly lower specific energy consumption even at operating points corresponding to near‐peak productivity. While the determination of the precise optimal adsorption time for real‐world deployment necessitates a comprehensive techno‐economic assessment, TPMS contactors offer a superior operational paradigm that synergistically achieves high productivity and enhanced energy efficiency.

The relatively high absolute energy consumption should be interpreted in the context of the current metallic prototype and conservative regeneration assumptions. The contactors were fabricated from AlSi10Mg with a wall thickness of approximately 150 µm to ensure reliable additive manufacturing of monoliths. This design introduces a substantial inactive thermal mass. To further clarify the origin of the thermal penalty associated with this metallic substrate, we performed a wall‐thickness sensitivity analysis by reducing the substrate wall thickness from 150 to 25 µm while keeping the adsorption capacity, kinetic parameters, and operating conditions unchanged (Figure 5d). This analysis isolates the contribution of the inactive metallic substrate to the sensible heat load. For the Gyroid contactor, the total energy consumption decreases from 11.90 to 10.59, 9.28, and 8.62 GJ/t_CO2_, respectively. For the Diamond contactor, the corresponding values decrease from 12.34 to 10.81, 9.28, and 8.52 GJ/t_CO2_. Although the square‐channel contactor also benefits from wall‐thickness reduction, the TPMS structures retain a clear relative advantage. Across the 150–25 µm wall‐thickness range, Gyroid exhibits 46.3%–51.7% lower total energy consumption than Square, while Diamond exhibits 46.9–49.9% lower total energy consumption. These results indicate that the high absolute energy consumption is largely associated with the current metal‐substrate prototype, whereas the relative energy‐saving advantage of TPMS originates from enhanced mass transfer and improved effective CO_2_ capture per cycle.

Lower‐heat‐capacity materials would further improve the energy performance of TPMS‐based contactors, while preserving the transport benefits associated with their tortuous architectures. Moreover, the adsorbent coating was kept 50 µm to isolate the hydrodynamic effect of contactor geometry, leading to a relatively low active‐sorbent fraction compared with optimized laminate, fiber, polymeric, or ceramic contactors [15, 17, 28, 58, 59]. To assess whether the present geometries remain advantageous relative to fiber sorbents (another important low‐resistance contactor class with a high active sorbent fraction and low inactive thermal mass [28, 29, 30]), we compared the two contactor families on a consistent basis using the validated performance model. Because fiber and TPMS contactors differ primarily in their active sorbent fraction and in whether they provide lateral flow mixing, we represented a fiber‐type contactor using the square‐channel mass‐transfer kinetics and pressure drop (an axial‐flow contactor without TPMS secondary‐flow mixing) evaluated in the zero‐inactive‐mass limit of the wall‐thickness analysis (Figure 5d). Under matched active sorbent loading and identical dry‐regeneration assumptions, this fiber‐type limit reaches 14.4 GJ/t_CO2_, whereas a porous‐wall TPMS contactor reaches 8.0 GJ/t_CO2_ in the same limit; even the current metallic Gyroid (11.9 GJ/t_CO2_) consumes less energy per ton of CO_2_ than the fiber‐type limit despite its full inactive‐mass and fan‐energy penalties. Under the present condition, the high active sorbent fraction represented by the fiber‐type axial‐flow limit lowers the sensible heat load but does not fully compensate for the utilization benefit provided by TPMS lateral mixing. The two effects are complementary that the active‐fraction benefit exploited by fiber sorbents and the lateral‐mixing benefit isolated here are most effective when combined in a high‐sorbent‐loaded TPMS contactor (Table S18).

It is important to delineate which of the present conclusions are expected to transfer to high‐loading contactors of TPMS structures. The enhancement mechanism identified here includes geometry‐induced chaotic advection, repeated flow splitting and recombination, and the consequent thinning and continuous renewal of the external concentration boundary layer. It operates entirely in the open gas channel and is therefore intrinsic to the flow topology, independent of how the sorbent is incorporated into the wall. These channel‐side benefits, together with the more uniform flow and purge‐gas distribution and the convective sweep advantage during regeneration, are expected to remain operative and transferable to TPMS contactors with higher adsorbent loadings. In contrast, the quantitative magnitudes reported here pertain to the present moderate adsorbent‐loading regime. As sorbent loading increases, intra‐wall diffusion grows in relative importance, as evidenced by the decrease of the fast‐site fraction *η* from 0.57 to 0.36 when the dense coating was thickened from Square to Square+. The TPMS advantage is therefore maximized when the contactor remains near the externally controlled mass‐transfer regime, which suggests a clear design rule. Higher sorbent loading should be pursued by increasing wall porosity and internal surface area while keeping the intra‐wall diffusion path short, rather than by thickening a dense, low‐diffusivity layer. Practical routes that follow this rule include impregnating sorbent into macroporous printed walls, directly fabricating sorbent‐rich TPMS bodies by templated phase inversion [53], functionally graded walls combining a thin high‐diffusivity skin with higher interior loading, and pairing these with low‐thermal‐mass materials and thinner walls (Figure 5d).

Because the transport enhancement in TPMS structures is accompanied by additional hydraulic resistance, the pressure‐drop characteristics were also compared (Figure 5e). As expected, the Square contactor exhibits the lowest pressure drop because of its straight channels, whereas the TPMS contactors show higher pressure drops due to flow redirection, secondary motion, and increased momentum dissipation at the curved walls. However, the pressure drops of the TPMS contactors remain substantially lower than that of a representative packed bed over the evaluated superficial‐velocity range. Among the TPMS geometries, Diamond exhibits the lowest hydraulic penalty, whereas Primitive shows the highest pressure drop. This is primarily because the Primitive structure has the smallest unit‐cell size under otherwise identical geometric constraints, which increases viscous dissipation and momentum loss, leading to a higher pressure drop. To explicitly quantify this hydraulic trade‐off, the fan‐energy‐normalized CO_2_ capture was also calculated from the fan‐energy contribution (Table S13). The straight‐channel Square contactor shows the highest CO_2_ captured per fan energy because of its minimal pressure drop, whereas TPMS contactors require higher fan energy due to flow redirection and secondary motion. Among the TPMS geometries, Diamond gives the most favorable hydraulic performance, with an Efan of 0.87 GJ/t_CO2_, compared with 1.45 and 2.22 GJ/t_CO2_ for Gyroid and Primitive, respectively. This hydraulic penalty has been explicitly included in the fan‐energy term of the total energy calculation in Figure 5c. Therefore, the lower total energy consumption of TPMS contactors is not obtained by neglecting pressure drop, but rather by balancing the additional fan energy against the larger amount of CO_2_ captured per cycle.

To conduct a multi‐dimensional evaluation of overall contactor performance, a radar chart analysis was employed (Figure 5f) based on six key performance indicators (KPIs): adsorption kinetics, adsorbent utilization rate, desorption kinetics, productivity, energy efficiency, and adsorption capacity. The quantitative data for each KPI was normalized based on results detailed in previous sections and summarized in Table S15 and Table S16.

The Square‐series comparison illustrates the intrinsic limitation of simply increasing adsorbent loading in straight channels. Square− exhibits relatively fast adsorption and desorption kinetics, but its low volumetric adsorption capacity leads to poor productivity and energy efficiency. Square+ improves volumetric capacity, but the thicker coating compromises kinetic accessibility and adsorbent utilization. In comparison, the TPMS contactors provide a more balanced performance profile by improving adsorption/desorption kinetics and adsorbent utilization while maintaining lower specific energy consumption. Among them, Gyroid and Diamond show the strongest combined performance, whereas Primitive provides an intermediate case. These results reinforce that the benefit of TPMS contactors arises from a coupled transport advantage rather than from a single metric alone.

Compared with the recently reported polymer–sorbent TPMS DAC contactors [53], which established the feasibility of complex PEI/silica TPMS geometries, the present study isolates the hydrodynamic contribution of TPMS topology by using geometrically matched metallic substrates with controlled coating mass. This enables a direct comparison among Square, Gyroid, Primitive, and Diamond contactors, linking flow topology to breakthrough kinetics, dual‐site accessibility, pressure drop, productivity, and energy consumption.

## 3 Conclusions

In this study, the critical trade‐off between adsorbent capacity and mass transfer kinetics of contactors in DAC was addressed. Through a systematic comparison of additively manufactured TPMS contactors and traditional monoliths with varying adsorbent loadings, results demonstrate that the engineered tortuosity of TPMS architectures is key to enhancing process efficiency. Superior adsorption/desorption kinetics, higher adsorbent utilization, and consequently, a substantial increase in CO_2_ productivity alongside a significant reduction in specific energy consumption are achieved by TPMS structures, particularly the Gyroid. This enhancement is mechanistically attributed to the ability of TPMS to address the flow field limitations ignored in conventional designs. By inducing sustained secondary flows and chaotic advection, these structures actively scour the adsorbent surface and minimize the laminar boundary layer thickness, effectively bridging the gap between fluid dynamics and chemical adsorption. An architecture‐driven design paradigm was established in which the contactor functions not as a passive scaffold for the adsorbent, but as an active hydrodynamic modulator. By demonstrating a pathway to alleviate the trade‐off between process kinetics and volumetric capacity, the focus of optimization is shifted toward the synergistic integration of materials and structural design. While a robust proof‐of‐concept was provided at the lab scale, the path to industrial deployment necessitates additional research. Future work should prioritize the techno‐economic analysis of scaled TPMS contactor manufacturing, the computational optimization of next‐generation TPMS geometries like functionally graded or multi‐scale structures, and the physical integration of thermal management functions directly within the contactor architecture. It should also be emphasized that the present productivity and energy analysis is based on dry conditions. H_2_O co‐adsorption/desorption and associated humid‐regeneration effects were not included, which considerably affect the energy efficiency and cycle productivity under practical DAC conditions. Therefore combining TPMS contactor design with water‐tolerant sorbent development and optimized regeneration processes like steam‐assisted TSA and TVSA should be studied to evaluate practical humid‐air DAC performance.

## 4 Methods

### 4.1 Contactor Fabrication

The square‐channel structures were designed using SolidWorks software. The TPMS structure was generated using the open‐source MATLAB plugin MSLattice [60]. As detailed in Table 1, key geometric parameters including wall thickness, relative density, specific surface area, and hydraulic diameter were matched between the Square and TPMS substrates. All substrates were cylindrical with a diameter of 18 mm and a height of 30 mm to fit the dimensions of the custom fixed‐bed reactor (Section 4.3) used for CO_2_ breakthrough experiments. The substrates were fabricated using aluminum alloy (AlSi10Mg) powder as the feedstock with laser powder bed fusion metal 3D printer.

Polyethylenimine‐functionalized mixed metal oxides (PEI/MMO) have emerged as a promising Class 1 DAC adsorbent [61, 62, 63, 64]. This composite is prepared by impregnating PEI onto layered double hydroxide‐derived mixed metal oxides (MMOs), where the defect‐abundant MMO layers provide strong electrostatic attraction to the polyamines [65]. This structure optimizes PEI distribution and CO_2_ accessibility, enabling the powder material to exhibit an unexpectedly large CO_2_ uptake of 2.27 mmol/g, fast kinetics, and high thermal and cyclic stability under ultradilute conditions [43, 61]. Material synthesis details are provided in the Section S1. The metal substrates were functionalized with PEI/MMO via a dip‐coating method in toluene solution with polystyrene‐block‐polybutadiene‐block‐polystyrene (SBS) as the binder. SBS is critical for enhancing the hydrothermal stability and moisture tolerance of the amine‐based adsorbent in practical applications [59], ensuring stable and uniform adhesion of the material particles to the metal substrate, and maintain the porous structure of the composite, facilitating favorable CO_2_ diffusion [32].

Typically, MMO powder, PEI, and SBS were added to toluene in a weight ratio of 3:6:1. The mass ratio of toluene to MMO was fixed at 8:1. The mixture was stirred for 3 h at 25°C to form a homogeneous suspension. The metal substrate was ultrasonically cleaned in ethanol for 10 min to remove surface impurities, then dried completely. The dry mass of the substrate was recorded (*m*_1_). The cleaned substrate was slowly immersed into the coating solution to ensure complete wetting of both the external surface and internal pores. After immersion, the substrate was carefully withdrawn. Excess coating solution blocking the pores was removed using 1 L/min flow of N_2_ for 1 min. The coated substrate was allowed to rest at 25°C for 5 min for initial solvent evaporation and shape setting. This sequence constituted one coating cycle. For the standard coating thickness, Square substrates were subjected to six coating cycles. The same number of cycles was applied to TPMS substrates to achieve comparable coating thickness. To investigate the effect of coating thickness on adsorption performance, additional Square substrates were coated for three cycles (denoted as Square−) and nine cycles (denoted as Square+). After the final coating cycle, the coated substrates were dried in a vacuum oven at 70 °C for at least 12 h to ensure complete removal of toluene. The mass of the coated and dried substrate was recorded (*m*_2_). The mass of the deposited coating was calculated as (*m*_2_ − *m*_1_).

### 4.2 Material Characterizations

The microstructure of the adsorbent coating was examined using a scanning electron microscope (SEM, RISE‐MAGNA, Tescan, Czech Republic). The skeletal density of the adsorbent coating material was determined using an Ultrapyc 5000 gas pycnometer (Anton Paar, USA). The thermal conductivity and specific heat capacity of the adsorbent coating material were measured using a laser flash analyzer (LFA 467, Netzsch, Germany).

The CO_2_ adsorption capacity and uptake rate of the adsorbent coating were measured using a thermogravimetric analyzer (TGA8000, PerkinElmer, USA). Prior to testing, the coating material scraped from the substrate was pretreated at 120°C for 2 h under a 90 mL/min flow of N_2_. The temperature was then lowered to the adsorption temperature of 25°C and stabilized for 1 h under the same N_2_ flow. Subsequently, the inlet gas was switched to a mixture of 400 ppm CO_2_ in N_2_ at a flow rate of 90 mL/min for 3 h to monitor the mass gain due to CO_2_ adsorption. The PEI content of the final coatings was quantified by thermogravimetric analysis. Coating samples were heated from 30 to 700°C under N_2_ at 20°C/min. CO_2_ adsorption isotherms were obtained using a BELSORP‐MAX analyzer (MicrotracBEL, Japan). Samples were degassed under vacuum at 100°C for 6 h prior to analysis. Adsorption measurements were conducted at 25, 45, and 65°C using CO_2_ as the adsorbate gas.

### 4.3 Breakthrough Experiments

CO_2_ breakthrough experiments were performed in a custom‐built fixed‐bed reactor (Figure S5) with an adsorption column inner diameter of 18 mm. Before each experiment, the bed containing the coated adsorbent monolith was regenerated by heating to 120 °C using a circulating oil bath while purging with N_2_ at a flow rate of 100 mL/min for 1 h to desorb any residual components. The N_2_ purge stream was also heated to the same temperature before entering the bed. The bed was then cooled to 25 °C. The adsorption step was initiated by introducing a feed gas mixture of 400 ppm CO_2_ in N_2_ at flow rates of 1 or 2 L/min for 3 h. The CO_2_ concentration at the outlet of the adsorption bed was continuously monitored during adsorption using a GXH‐3011N online infrared gas analyzer (Huayun Instruments, China; range: 0^−1000^ ppm; linear error ≤ ± 2% of full scale). The composition of the gas stream during the subsequent desorption step was monitored at the desorption outlet using an A‐CMI420 gas detection system (Vaisala, Finland; range: 0%–1%, 1%–20%). Prior to breakthrough experiments, a dead volume correction was performed using a blank column (no sorbent) at each flow rate to determine the system void volume and piping residence time. The breakthrough time and adsorption capacity calculations have been corrected accordingly. Details including the blank column breakthrough time of 1 and 2 L/min and the result dead volume correction applied to each reported breakthrough curve have been added to Figure S8.

Each breakthrough curve corresponds to a single measurement for a given contactor and flow rate. Reproducibility was assessed through triplicate sample fabrication and coating‐mass measurement (relative deviation ≤1.86%, Table S2). The composite measurement uncertainty was estimated at ≈ ± 5.5% by propagating the infrared analyzer linear error (≤ ± 2% full scale), the mass flow controller accuracy (± 1%), and the dead‐volume correction (<2%, Figure S8) in quadrature.

### 4.4 Modeling Methodology

A comprehensive modeling framework was developed to simulate the CO_2_ adsorption process in the fixed‐bed reactor loaded with structured adsorbent monoliths. The model integrates an adsorption equilibrium isotherm, adsorption kinetics model, and a fixed‐bed reactor model, as detailed in Section S3.

### 4.5 Adsorption Equilibrium Model

The equilibrium adsorption behavior of CO_2_ on the adsorbent was described using the Langmuir isotherm model, which assumes monolayer adsorption on a homogeneous surface (Equation (1)).

(1) $$q_{e}=\frac{q_{m}k_{L}P_{CO_{2}}}{1+k_{L}P_{CO_{2}}}\;$$

Where *q*_e_ is the equilibrium adsorption capacity (mol·kg^−^^1^), *q_m_* is the theoretical monolayer saturation capacity (mol·kg^−^^1^), *k*_L_ is the temperature‐dependent equilibrium constant (Pa^−^^1^), and *P* is the partial pressure of CO_2_ (Pa).

The temperature dependence of the equilibrium constant *k*_L_ follows an Arrhenius‐type expression (Equation (2)).

(2) $$k_{L}=k_{0}exp\left(-\frac{E}{RT}\right)$$

Where *k*_0_ is the pre‐exponential factor (Pa^−^^1^), *E* is the adsorption energy (J/mol), *R* is the universal gas constant (8.314 J/mol/K), and *T* is the absolute temperature (K).

The model parameters (*q_m_*, *k*_L_, and *E*) were determined by fitting Eq. (2) to the CO_2_ adsorption isotherm data measured at 298.15, 318.15, and 338.15 K using BELSORP‐MAX analyzer. The fitted parameter values are reported in Table S5 and fitting results are shown in Figure S11.

Adsorption kinetics model. Adsorption kinetics were initially modelled using the linear driving force (LDF) approximation, which assumes the adsorption rate is proportional to the difference between the equilibrium loading and the instantaneous loading (Eq. (3)).

(3) $$\frac{dq}{dt}=k\times \left(q_{e}-q\right)$$

Where *q* is the adsorbed amount at time *t* and *k* is the overall mass transfer coefficient (s^−1^).

An initial estimate for *k* (3.73×10^−4^ s^−1^) was obtained by fitting Eq. (3) to the TGA uptake data. This suggested limitations of the single‐rate LDF model, likely arising from kinetic heterogeneity within the adsorbent coating. To account for this, a dual‐resistance LDF model was implemented, considering distinct mass transfer pathways. The specific formulas for the DK model are listed in Eq. (4) and Eq. (5).

(4) $$\begin{array}{c} \;\frac{\partial q_{1}}{\partial t}=k_{1}\;\left(\eta q_{e}-q_{1}\right) \end{array}$$

(5) $$\begin{array}{c} \;\frac{\partial q_{2}}{\partial t}=k_{2}\;\left(\left(1-\eta \right)q_{e}-q_{2}\right) \end{array}$$

where q1 and q2 are the amount adsorbed on the fast and slow adsorption sites, respectively. The parameter *η* represents the fraction of fast adsorption sites relative to the total number of adsorption sites. *k*_1_ and *k*_2_ denote the mass transfer coefficient from the gas phase to the fast adsorption sites, and then to the slow adsorption sites. Both *k*_1_ and *k*_2_ are apparent lumped mass‐transfer coefficients comprising the external gas‐film resistance in series with the respective internal (coating‐side) resistance.

Fixed‐bed reactor model. A one‐dimensional, non‐isothermal, and non‐adiabatic fixed‐bed model was developed to simulate the transport phenomena during CO_2_ adsorption in the monolithic honeycomb contactor. The model equations presented in Table S6 consist of mass balance accounting for axial dispersion, convection, and adsorption source terms coupled through the dual‐LDF kinetics; momentum balance described by a pressure drop correlation of the Hagen‐Poiseuille equation adapted for the honeycomb geometry; and energy balance that incorporates convective heat transfer, axial conduction, heat of adsorption/desorption, and heat exchange with the reactor wall. The associated boundary conditions are provided in Table S7.

### 4.6 Computational Fluid Dynamics Simulations

Pressure drops of the structured contactors were obtained from computational fluid dynamics simulations and reported as pressure drop per unit length. The computational modeling was performed using COMSOL Multiphysics. The CFD pressure‐drop trends were validated by scaled experimental measurements under Reynolds‐number similarity (Figure S17). For comparison, the pressure drop of a representative packed bed was calculated using the Ergun equation, as detailed in the Section S4.

### 4.7 Performance Evaluation

A scaled‐up contactor model with a length of 300 mm was utilized for performance evaluation, under the assumption of fully developed axial flow where adsorption behavior scales linearly with bed length, neglecting entrance/exit effects which are minimized in high‐aspect‐ratio industrial beds. Detailed contactor parameters are provided in Table S14. The performance of the adsorbent contactor was evaluated using the following KPIs calculated from the adsorption cycle: Specific productivity (*P*, mol_CO2_/m^3^/d, Equation S5) and total specific energy consumption (*W*_total_, GJ/t_CO2_). The total energy consumption comprises sensible heat required to heat the adsorbent and contactor structure (*E*_sensible_heat_), sensible heat required to heat the nitrogen purge gas (*E*_purge_), heat of desorption for CO_2_ (*E*_adsorption_heat_), and energy consumed by fans for gas flow (*E*_fan_). Detailed calculation methods and parameters used for the KPI evaluation are provided in the Section S5.

A wall‐thickness sensitivity analysis was conducted to evaluate the contribution of the metallic substrate to the total sensible heat load. The substrate wall thickness was varied from 150 to 100, 50, and 25 µm. In this analysis, the adsorption capacity, fitted kinetic parameters, pressure drop, and cycle conditions were kept unchanged, while the substrate mass was scaled according to the wall thickness.

## Author Contributions

**Zhuozhen Gan**: methodology, data curation, investigation, writing – review and editing, writing – original draft. **Qingyang Shao**: methodology, data curation, investigation, visualization, writing – review and editing, writing – original draft, conceptualization. **Xuancan Zhu**: visualization, writing – review and editing, supervision, conceptualization. **Chengcheng Long**: writing – review and editing, software. **Man Zhang**: writing – review and editing. **Yuehui Li**: writing – review and editing, supervision. **Yihe Miao**: writing – review and editing.

## Conflicts of Interest

There are no conflicts of interest to declare.

## Supporting information




**Supporting File**: advs76487‐sup‐0001‐SuppMat.docx.

## Data Availability

All data which support this study are included in the published Article and its supplementary information.

## References

[1] M. Erans , E. S. Sanz‐Pérez , D. P. Hanak , Z. Clulow , D. M. Reiner , and G. A. Mutch , “Direct Air Capture: Process Technology, Techno‐Economic and Socio‐Political Challenges,” Energy & Environmental Science 15, no. 4 (2022): 1360–1405, 10.1039/D1EE03523A.

[2] G. Realmonte , L. Drouet , A. Gambhir , et al., “An Inter‐Model Assessment of the Role of Direct Air Capture in Deep Mitigation Pathways,” Nature Communications 10, no. 1 (2019): 3277, 10.1038/s41467-019-10842-5. PMC6646360 31332176

[3] J. Meckling and E. Biber , “A Policy Roadmap for Negative Emissions Using Direct Air Capture,” Nature Communications 12, no. 1 (2021): 2051, 10.1038/s41467-021-22347-1. PMC8024265 33824338

[4] X. Zhu , W. Xie , J. Wu , et al., “Recent Advances in Direct Air Capture by Adsorption,” Chemical Society Reviews 51, no. 15 (2022): 6574–6651, 10.1039/D1CS00970B. 35815699

[5] D. W. Keith , G. Holmes , D. Angelo , and K. Heidel , “A Process for Capturing CO2 from the Atmosphere,” Joule 2, no. 8 (2018): 1573–1594, 10.1016/j.joule.2018.05.006.

[6] H. Li , M. E. Zick , T. Trisukhon , et al., “Capturing Carbon Dioxide from Air with Charged‐Sorbents,” Nature 630, no. 8017 (2024): 654–659, 10.1038/s41586-024-07449-2. PMC11186774 38839965

[7] S. Mukherjee , N. Sikdar , D. O'Nolan , et al., “Trace CO2 Capture by an Ultramicroporous Physisorbent with Low Water Affinity,” Science Advances 5, no. 11 (2019): aax9171, 10.1126/sciadv.aax9171. PMC6884411 31819904

[8] K. Madhu , S. Pauliuk , S. Dhathri , and F. Creutzig , “Understanding Environmental Trade‐Offs and Resource Demand of Direct Air Capture Technologies through Comparative Life‐Cycle Assessment,” Nature Energy 6, no. 11 (2021): 1035–1044, 10.1038/s41560-021-00922-6.

[9] Z. Zhou , T. Ma , H. Zhang , et al., “Carbon Dioxide Capture from Open Air Using Covalent Organic Frameworks,” Nature 635, no. 8037 (2024): 96–101, 10.1038/s41586-024-08080-x. 39443804

[10] B. R. Sutherland , “Pricing CO2 Direct Air Capture,” Joule 3, no. 7 (2019): 1571–1573, 10.1016/j.joule.2019.06.025.

[11] H. Chen , H. Dong , Z. Shi , and A. K. SenGupta , “Direct Air Capture (DAC) and Sequestration of CO2: Dramatic Effect of Coordinated Cu(II) onto a Chelating Weak Base Ion Exchanger,” Science Advances 9, no. 10 (2023): adg1956, 10.1126/sciadv.adg1956. PMC9995034 36888712

[12] M. Poderyte , R. Lima , P. I. Golbækdal , et al., “Repurposing Polyethylene Terephthalate Plastic Waste to Capture Carbon Dioxide,” Science Advances 11, no. 36 (2025): adv5906, 10.1126/sciadv.adv5906. PMC12412650 40911693

[13] Y. Miao , Y. Wang , B. Ge , et al., “Mixed Diethanolamine and Polyethyleneimine with Enhanced CO2 Capture Capacity from Air,” Advanced Science 10, no. 16 (2023): 2207253, 10.1002/advs.202207253. PMC10238199 37017566

[14] J. Wu , X. Zhu , F. Yang , R. Wang , and T. Ge , “Shaping Techniques of Adsorbents and Their Applications in Gas Separation: A Review,” Journal of Materials Chemistry A 10 (2022): 22853–22895, 10.1039/D2TA04352A.

[15] Y. J. Min , A. Ganesan , M. J. Realff , and C. W. Jones , “Direct Air Capture of CO _2_ Using Poly(Ethyleneimine)‐Functionalized Expanded Poly(Tetrafluoroethylene)/Silica Composite Structured Sorbents,” ACS Applied Materials & Interfaces 14, no. 36 (2022): 40992–41002, 10.1021/acsami.2c11143. 36047596

[16] K. Sievert , T. S. Schmidt , and B. Steffen , “Considering Technology Characteristics to Project Future Costs of Direct Air Capture,” Joule 8, no. 4 (2024): 979–999, 10.1016/j.joule.2024.02.005.

[17] R. Wu , H. E. Delgado , Y. Xie , et al., “Distributed Direct Air Capture by Carbon Nanofiber Air Filters,” Science Advances 11, no. 42 (2025): adv6846, 10.1126/sciadv.adv6846. PMC12533599 41105774

[18] X. Sun , X. Shen , H. Wang , et al., “Atom‐Level Interaction Design between Amines and Support for Achieving Efficient and Stable CO_2_ Capture,” Nature Communications 15, no. 1 (2024): 5068, 10.1038/s41467-024-48994-8. PMC11176289 38871697

[19] M. Zhao , L. Huang , Y. Gao , et al., “Innovative Design of PEI‐Modified AMO‐Layered Double Hydroxide for Efficient and Stable Direct Air Capture of CO2,” Advanced Science 12, no. 37 (2025): 07756, 10.1002/advs.202507756. PMC12499402 40588786

[20] B. Verougstraete , A. Martín‐Calvo , S. Van Der Perre , G. Baron , V. Finsy , and J. F. M. Denayer , “A New Honeycomb Carbon Monolith for CO2 Capture by Rapid Temperature Swing Adsorption Using Steam Regeneration,” Chemical Engineering Journal 383 (2020): 123075, 10.1016/j.cej.2019.123075.

[21] I. Cornejo , P. Nikrityuk , and R. E. Hayes , “Heat and Mass Transfer inside of a Monolith Honeycomb: From Channel to Full Size Reactor Scale,” Catalysis Today 383 (2022): 110–122, 10.1016/j.cattod.2020.10.036.

[22] S. Chen , W. K. Shi , J. Y. Yong , et al., “Numerical Study on a Structured Packed Adsorption Bed for Indoor Direct Air Capture,” Energy 282 (2023): 128801, 10.1016/j.energy.2023.128801.

[23] J. Valentine and A. Zoelle , “Direct Air Capture Case Studies: Sorbent System,” in Direct air capture case studies: Sorbent system (National Energy Technology Laboratory, 2022).

[24] N. S. Borker , T. Herdtle , C. Sauer , I. H. Romdhane , and M. Pekurovsky , “Engineering Design of Direct‐Air‐Capture Contactors Composed of Sorbent Particles Using Numerical Simulations,” AIChE Journal 70, no. 2 (2024): 18303, 10.1002/aic.18303.

[25] V. Stampi‐Bombelli , A. Storione , Q. Grossmann , and M. Mazzotti , “On Comparing Packed Beds and Monoliths for CO2 Capture from Air through Experiments, Theory, and Modeling,” Industrial & Engineering Chemistry Research 63, no. 26 (2024): 11637–11653, 10.1021/acs.iecr.4c01392. PMC11228921 38983186

[26] M. Karimi , J. A. C. Silva , C. N. P. Gonçalves , J. L. Diaz de Tuesta , A. E. Rodrigues , and H. T. Gomes , “CO_2_ Capture in Chemically and Thermally Modified Activated Carbons Using Breakthrough Measurements: Experimental and Modeling Study,” Industrial & Engineering Chemistry Research 57, no. 32 (2018): 11154–11166, 10.1021/acs.iecr.8b00953.

[27] J. Wu , Y. Chen , K. Wang , et al., “Rapid Steam‐Assisted Temperature Swing Adsorption for Direct Air Capture Using a Rotary Adsorber,” Advanced Science 13, no. 19 (2026): 21499, 10.1002/advs.202521499. PMC13045267 41580976

[28] T. Femmer , A. J. C. Kuehne , and M. Wessling , “Estimation of the Structure Dependent Performance of 3‐D Rapid Prototyped Membranes,” Chemical Engineering Journal 273 (2015): 438–445, 10.1016/j.cej.2015.03.029.

[29] W. H. Lee , X. Zhang , S. Banerjee , C. W. Jones , M. J. Realff , and R. P. Lively , “Sorbent‐Coated Carbon Fibers for Direct Air Capture Using Electrically Driven Temperature Swing Adsorption,” Joule 7, no. 6 (2023): 1241–1259, 10.1016/j.joule.2023.05.016.

[30] J. Marreiros , Y. Wang , M. Song , et al., “Fiber Sorbents – A Versatile Platform for Sorption‐Based Gas Separations,” Accounts of Materials Research 6, no. 1 (2025): 6–16, 10.1021/accountsmr.4c00201. PMC11773446 39882339

[31] V. Stampi‐Bombelli and M. Mazzotti , “Exploring Geometric Properties and Cycle Design in Packed Bed and Monolith Contactors Using Temperature‐Vacuum Swing Adsorption Modeling for Direct Air Capture,” Industrial & Engineering Chemistry Research 63, no. 45 (2024): 19728–19743, 10.1021/acs.iecr.4c02303. PMC11565576 39553914

[32] Y. Seok Chae , S. Park , D. W. Kang , et al., “Moisture‐Tolerant Diamine‐Appended Metal–Organic Framework Composites for Effective Indoor CO2 Capture through Facile Spray Coating,” Chemical Engineering Journal 433 (2022): 133856, 10.1016/j.cej.2021.133856.

[33] Q. Grossmann , V. Stampi‐Bombelli , A. Yakimov , S. Docherty , C. Copéret , and M. Mazzotti , “Developing Versatile Contactors for Direct Air Capture of CO2 through Amine Grafting onto Alumina Pellets and Alumina Wash‐Coated Monoliths,” Industrial & Engineering Chemistry Research 62, no. 34 (2023): 13594–13611, 10.1021/acs.iecr.3c01265. PMC10472440 37663169

[34] E. Tegeler , Y. Cui , M. Masoudi , et al., “A Novel Contactor for Reducing the Cost of Direct Air Capture of CO2,” Chemical Engineering Science 281 (2023): 119107, 10.1016/j.ces.2023.119107.

[35] I. Ziółkowska and D. Ziółkowski , “Modelling of Gas Interstitial Velocity Radial Distribution over Cross‐Section of a Tube Packed with Granular Catalyst Bed; Effects of Granule Shape and of Lateral Gas Mixing,” Chemical Engineering Science 62, no. 9 (2007): 2491–2502, 10.1016/j.ces.2007.01.029.

[36] G. Zhang , P. Zhao , L. Hao , Y. Xu , and H. Cheng , “A Novel Amine Double Functionalized Adsorbent for Carbon Dioxide Capture Using Original Mesoporous Silica Molecular Sieves as Support,” Separation and Purification Technology 209 (2019): 516–527, 10.1016/j.seppur.2018.07.074.

[37] J. A. Wurzbacher , C. Gebald , S. Brunner , and A. Steinfeld , “Heat and Mass Transfer of Temperature–Vacuum Swing Desorption for CO2 Capture from Air,” Chemical Engineering Journal 283 (2016): 1329–1338, 10.1016/j.cej.2015.08.035.

[38] S. Lawson , B. Adebayo , C. Robinson , Q. Al‐Naddaf , A. A. Rownaghi , and F. Rezaei , “The Effects of Cell Density and Intrinsic Porosity on Structural Properties and Adsorption Kinetics in 3D‐Printed Zeolite Monoliths,” Chemical Engineering Science 218 (2020): 115564, 10.1016/j.ces.2020.115564.

[39] O. I.‐F. Chen , C.‐H. Liu , K. Wang , et al., “Water‐Enhanced Direct Air Capture of Carbon Dioxide in Metal–Organic Frameworks,” Journal of the American Chemical Society 146, no. 4 (2024): 2835–2844, 10.1021/jacs.3c14125. 38236722

[40] J. Yu , J. Zhu , L. Chen , Y. Chao , W. Zhu , and Z. Liu , “A Review of Adsorption Materials and Their Application of 3D Printing Technology in the Separation Process,” Chemical Engineering Journal 475 (2023): 146247, 10.1016/j.cej.2023.146247.

[41] A. Pereira , A. F. P. Ferreira , A. E. Rodrigues , A. M. Ribeiro , and M. J. Regufe , “Additive Manufacturing for Adsorption‐related Applications—A Review,” Journal of Advanced Manufacturing and Processing 4, no. 1 (2022): 10108, 10.1002/amp2.10108.

[42] X. Li , H. Thakkar , A. A. Rownaghi , and F. Rezaei , “Recent Advances in 3D Printing of Structured Materials for Adsorption and Catalysis Applications,” Chemical Reviews 121, no. 10 (2021): 6246–6291, 10.1021/acs.chemrev.1c00060. 33947187

[43] Q. Shao , Z. Gan , B. Ge , et al., “3D Printing of Poly(Ethyleneimine)‐Functionalized Mg‐al Mixed Metal Oxide Monoliths for Direct Air Capture of CO2,” Journal of Energy Chemistry 96 (2024): 491–500, 10.1016/j.jechem.2024.05.015.

[44] H. Thakkar , S. Eastman , A. Hajari , A. A. Rownaghi , J. C. Knox , and F. Rezaei , “3D‐Printed Zeolite Monoliths for CO2 Removal from Enclosed Environments,” ACS Applied Materials & Interfaces 8, no. 41 (2016): 27753–27761, 10.1021/acsami.6b09647. 27658639

[45] H. Thakkar , S. Eastman , A. Al‐Mamoori , A. Hajari , A. A. Rownaghi , and F. Rezaei , “Formulation of Aminosilica Adsorbents into 3D‐Printed Monoliths and Evaluation of Their CO _2_ Capture Performance,” ACS Applied Materials & Interfaces 9, no. 8 (2017): 7489–7498, 10.1021/acsami.6b16732. 28186400

[46] S. M. Lawson , (Missouri University of Science and Technology ProQuest Dissertations & Theses, 2021): 28414782.

[47] L. F. A. S. Zafanelli , A. Henrique , H. Steldinger , et al., “3D‐Printed Activated Carbon for Post‐Combustion CO2 Capture,” Microporous and Mesoporous Materials 335 (2022): 111818, 10.1016/j.micromeso.2022.111818.

[48] A. Pereira , A. F. P. Ferreira , A. Rodrigues , A. M. Ribeiro , and M. J. Regufe , “Evaluation of the Potential of a 3D‐Printed Hybrid Zeolite 13X/Activated Carbon Material for CO2/N2 Separation Using Electric Swing Adsorption,” Chemical Engineering Journal 450 (2022): 138197, 10.1016/j.cej.2022.138197.

[49] J. Feng , J. Fu , X. Yao , and Y. He , “Triply Periodic Minimal Surface (TPMS) Porous Structures: From Multi‐Scale Design, Precise Additive Manufacturing to Multidisciplinary Applications,” International Journal of Extreme Manufacturing 4, no. 2 (2022): 022001, 10.1088/2631-7990/ac5be6.

[50] N. C. Ellebracht , P. Roy , T. Moore , et al., “3D Printed Triply Periodic Minimal Surfaces as Advanced Structured Packings for Solvent‐Based CO2 Capture,” Energy & Environmental Science 16, no. 4 (2023): 1752–1762, 10.1039/D2EE03658D.

[51] M. G. Gado and S. Ookawara , “3D‐Printed Triply Periodic Minimal Surface (TPMS) Structures: Towards Potential Application of Adsorption‐Based Atmospheric Water Harvesting,” Energy Conversion and Management 297 (2023): 117729, 10.1016/j.enconman.2023.117729.

[52] M. G. Gado , S. Ookawara , S. Nada , M. F. Elkady , and H. Hassan , “Adsorbent Beds Packed in Triply Periodic Minimal Surface‐Derived Structures and Their Performance in Adsorption Desalination/Cooling Systems,” International Communications in Heat and Mass Transfer 150 (2024): 107205, 10.1016/j.icheatmasstransfer.2023.107205.

[53] S. Kim , H. E. Holmes , Y. Wang , S. C. Weston , and R. P. Lively , “Polymer‐sorbent Direct Air Capture Contactors with Complex Geometries 3D‐printed via Templated Phase Inversion,” Advanced Functional Materials 35, no. 9 (2024): 2410356, 10.1002/adfm.202410356.

[54] H. Xu , Y. Zhang , Y. Mei , et al., “Hierarchical Sheet Triply Periodic Minimal Surface Lattices: Design, Performance and Optimization,” Applied Thermal Engineering 261 (2025): 125187, 10.1016/j.applthermaleng.2024.125187.

[55] Z. Zhang , T. Gao , B. Zhang , et al., “Conjugate Thermo‐Hydraulic Evaluation of Triply Periodic Minimal Surfaces and Pin Fins,” Applied Thermal Engineering 274 (2025): 126667, 10.1016/j.applthermaleng.2025.126667.

[56] J. Wang , K. Sun , M. Zeng , Q. Wang , and Z. Cheng , “Investigation on Uneven Flow Distribution in Triply Periodic Minimal Surface Heat Exchangers,” Energy Conversion and Management 314 (2024): 118713, 10.1016/j.enconman.2024.118713.

[57] F. L. Rashid , N. M. L. Al Maimuri , M. A. Al‐Obaidi , et al., “Enhancing Heat Transfer across Applications with Triply Periodic Minimal Surface (TPMS) Structures: A Comprehensive Review,” Chemical Engineering and Processing – Process Intensification 216 (2025): 110460, 10.1016/j.cep.2025.110460.

[58] Y. J. Min , J. Kim , C. W. Jones , and M. J. Realff , “Model‐Based Energy and Cost Analysis of Direct Air Capture Using ePTFE‐Based Laminate‐Structured Gas–Solid Contactors,” ACS Sustainable Chemistry & Engineering 12, no. 52 (2024): 18522–18536, 10.1021/acssuschemeng.4c05769.

[59] J. Wu , Y. Chen , Y. Xu , et al., “Facile Synthesis of Structured Adsorbent with Enhanced Hydrophobicity and Low Energy Consumption for CO2 Capture from the Air,” Matter 7, no. 1 (2024): 123–139, 10.1016/j.matt.2023.10.019.

[60] O. Al‐Ketan and R. K. Abu Al‐Rub , “MSLattice: A Free Software for Generating Uniform and Graded Lattices Based on Triply Periodic Minimal Surfaces,” Material Design & Processing Communications 3, no. 6 (2021): 205, 10.1002/mdp2.205.

[61] X. Zhu , T. Ge , F. Yang , et al., “Efficient CO2 Capture from Ambient Air with Amine‐Functionalized Mg–Al Mixed Metal Oxides,” Journal of Materials Chemistry A 8, no. 32 (2020): d0ta05079b, 10.1039/D0TA05079B.

[62] X. Zhu , M. Lyu , T. Ge , et al., “Modified Layered Double Hydroxides for Efficient and Reversible Carbon Dioxide Capture from Air,” Cell Reports Physical Science 2, no. 7 (2021): 100484, 10.1016/j.xcrp.2021.100484.

[63] B. Ge , C. Chen , Y. Xu , et al., “Enhancing Adsorbent Performance for Direct Air Capture of CO2 by In‐Situ Amine‐Grafting of Layered Double Hydroxides,” Chemical Engineering Journal 500 (2024): 156782, 10.1016/j.cej.2024.156782.

[64] Z. Gan , Q. Shao , B. Ge , Q. Wang , and X. Zhu , “Single‐Component and Binary H2O and CO2 Co‐Adsorption Isotherm Model on Amine‐Functionalised Mg‐al Mixed Metal Oxides,” Carbon Capture Science & Technology 14 (2025): 100328, 10.1016/j.ccst.2024.100328.

[65] M. Zhao , J. Xiao , W. Gao , and Q. Wang , “Defect‐Rich Mg‐al MMOs Supported TEPA with Enhanced Charge Transfer for Highly Efficient and Stable Direct Air Capture,” Journal of Energy Chemistry 68 (2022): 401–410, 10.1016/j.jechem.2021.12.031.

