# Supplementary material for: Efficient Adsorption‐Based Direct Air Capture Via Triply Periodic Minimal Surface Architectures
Source: Adv Sci (Weinh). 2026 Jul 8;13(55):e76487. doi: 10.1002/advs.76487 (PMC13344064; doi:10.1002/advs.76487)
Supplement: Supplementary file 1 [file ADVS-13-e76487-s001.docx]

Supplementary information

**Efficient adsorption-based direct air capture via triply periodic minimal surface** **architectures**

Qingyang Shao †^a,b^, Zhuozhen Gan †^a^, Chengcheng Long ^a^, Man Zhang ^a^, Yihe Miao ^b^, Yuehui Li ^b,*^, Xuancan Zhu ^a,*^

^a^ Research Center of Solar Power & Refrigeration, School of Mechanical Engineering, Shanghai Jiao Tong University, 800 Dongchuan Road, Shanghai, 200240, China

^b^ College of Smart Energy, Shanghai Jiao Tong University, 800 Dongchuan Road, Shanghai, 200240, China

† Authors contributed equally.

*Corresponding authors: E-mail:

[zhuxc19@sjtu.edu.cn](mailto:zhuxc19@sjtu.edu.cn) (X.C. Zhu)

[liyuehui@sjtu.edu.cn](mailto:liyuehui@sjtu.edu.cn) (Y.H. Li)

Content

[Section 1 Materials synthesis and characterization	3](#_Toc232347724)

[Section 2 Breakthrough experiments	9](#_Toc232347725)

[Section 3 Modeling adsorption	22](#_Toc232347726)

[Section 4 Pressure drop	34](#_Toc232347727)

[Section 5 Performance evaluation	42](#_Toc232347728)

[Reference	48](#_Toc232347729)

# Section 1 Materials synthesis and characterization

**Chemicals**

Mg(NO_3_)_2_·6H_2_O, Al(NO_3_)_3_·9H_2_O, NaOH were purchased from Macklin; Na_2_CO_3_ and acetone were purchased from Sinopharm Chemical Reagent; branched polyethyleneimine (PEI, M.W.~600, 98%, Gobekie), polystyrene-block-polybutadiene-block-polystyrene (SBS, 30 wt.% styrene, M.W.~140,000, Aladdin), and toluene (≥99.5%, General-reagent) were also obtained. All chemicals were used as received without further purification. Deionized (DI) water was used throughout all experiments.

**Adsorbent powder synthesis**

1) Synthesis of Mg-Al layered double hydroxides (LDH, Mg_0.55_Al-OH-CO_3_)

The LDH was synthesized following a previously reported co-precipitation method^1^. Briefly, 500 mL of an aqueous solution containing Mg(NO_3_)_2_·6H_2_O (0.55 mol L^−1^) and Al(NO_3_)_3_·9H_2_O (1 mol L^−1^) was prepared. Separately, 500 mL of Na_2_CO_3_ solution (0.5 mol L^−1^) and a 4 mol L^−1^ NaOH solution were prepared. The pH of the Na_2_CO_3_ solution was adjusted to 10.0 by dropwise addition of dilute HCl. The Mg-Al nitrate solution then slowly added to the Na_2_CO_3_ solution under vigorous stirring. Simultaneously, the NaOH solution was added dropwise to maintain the pH at 10.0 ± 0.2. The resulting slurry was stirred and aged at 25 °C for 16 hours. The precipitate was collected by filtration and washed thoroughly with DI water until the filtrate pH reached 7.0. The wet filter cake was subsequently washed with acetone for 4 hours. Finally, the sample was dried in a vacuum oven at 60 °C for 12 hours to obtain the LDH powder.

2) Synthesis of Mg-Al mixed metal oxide (MMO, Mg_0.55_Al-O)

The MMO was prepared by calcining the LDH precursor. The LDH powder was heated at a heating rate of 5 °C min^−1^ and kept at 450 °C for 6 hours under a continuous N_2_ flow to obtain the MMO powder.

Supplementary Fig. 1 Scanning electron microscope (SEM) images of adsorbent coating (Film-SBS-PEI/MMO).

Supplementary Fig. 2 Scanning electron microscope (SEM) images of adsorbent powder (PEI/MMO)^1^.

Supplementary Fig. 3 N_2_ adsorption isotherm and pore size distribution of adsorbent coating at 77K.

Supplementary Table 1. Textural properties of adsorbent

| Sample | Specific surface area  (m^2^ g^−1^) | Pore volume  (cm^3^ g^−1^) | Nominal PEI loading  (wt.%) | Adsorption capacity  (mmol g^−1^) |
| --- | --- | --- | --- | --- |
| Powder-MMO | 279.8 | 1.32 | 0 | − |
| Powder-PEI/MMO | 6.0 | 0.012 | 67 | 2.34 |
| Film-SBS-PEI/MMO | 3.3 | 0.012 | 60 | 1.88 |

The PEI loading values are nominal values calculated from the feed composition. The Powder-PEI/MMO sample was prepared using an MMO:PEI mass ratio of 3:6, giving a nominal PEI loading of 67 wt.%. The Film-SBS-PEI/MMO sample was prepared using an MMO:PEI:SBS mass ratio of 3:6:1, giving a nominal PEI loading of 60 wt.%. The reproducibility of the coating process is instead reflected by the three replicate coating masses reported in Supplementary Table 3.

**Supplementary Fig. 4** TGA quantification of the PEI content. TG (a) and DTG (b) curves of the PEI/MMO sorbent powder and of coating samples taken from the Square and Gyroid contactors (30–700 °C, N_2_, 20 °C/min). The first weight-loss step (≤150–170 °C) corresponds to desorption of pre-adsorbed H_2_O and CO_2_ and reflects sample storage history; PEI decomposition is assigned to the window between the dry point and the DTG valley at ≈470 °C, SBS decomposition to ≈470–600 °C, and the final residue to the MMO support. On a dry basis, the measured PEI contents are 66.8 wt% for the powder (nominal 67 wt%), and 61.5 wt% and 60.1 wt% for the Square and Gyroid coatings, respectively.

# Section 2 Breakthrough experiments

Supplementary Fig. 5 Scheme of custom-built fixed-bed reactor.

Supplementary Fig. 6 Images of Square contactors before and after coating.

Supplementary Fig. 7 Images of TPMS contactors before and after coating.

Supplementary Fig. 8 CO_2_ breakthrough curves in the blank experiment

As shown in Supplementary Fig. 8, the blank breakthrough curves obtained using the blank column rapidly reached the 95% inlet CO_2_ concentration at both 1 and 2 L min^-1^ with in 1 min, indicating that the contribution of system dead volume and non-adsorptive gas-phase holdup was limited. The blank curves were integrated using the same procedure as that used for the adsorption experiments. After calculation, the error in CO_2_ adsorption amount caused by the dead volume is within 2%. Therefore, the dead-volume correction only slightly shifted the absolute breakthrough times and had a negligible influence on the calculated adsorption capacities and the relative performance comparison among different contactor.

Supplementary Fig. 9 a, Breakthrough curves of square contactors with different coating mass, b Breakthrough curves of contactors with different structures.

Supplementary Table 2. Coating mass of contactors with different structures.

| Contactors | Coating mass (g) | | | | |  | Reference thickness  (μm) |
| --- | --- | --- | --- | --- | --- | --- | --- |
|  | Sample 1 | Sample 2 | Sample 3 | Average | Error | Relative mass |  |
| Square− | 0.185 | 0.179 | 0.187 | 0.184 | 0.42% | − | 14.1 |
| Square | 0.691 | 0.703 | 0.681 | 0.692 | 1.10% | 0% | 53.1 |
| Square+ | 1.161 | 1.169 | 1.152 | 1.161 | 0.85% | − | 89.1 |
| Gyroid | 0.708 | 0.716 | 0.695 | 0.706 | 1.06% | 2.02% | 54.2 |
| Primitive | 0.665 | 0.675 | 0.701 | 0.680 | 1.86% | 1.73% | 52.2 |
| Diamond | 0.693 | 0.707 | 0.684 | 0.695 | 1.16% | 0.43% | 53.3 |

$Thickness=\frac{Mass}{Density\times SurfaceArea}$	$(S1)$

Micro-computed tomography was considered as a possible method to evaluate the coating distribution. However, it was not used for quantitative coating-thickness analysis in this study because the PEI/MMO–SBS adsorbent layer is relatively thin, with a reference thickness of approximately 52–54 μm on the TPMS contactors, and is deposited on a highly X-ray-attenuating AlSi10Mg metallic substrate. Reliable micro-CT segmentation requires sufficient X-ray attenuation contrast between phases, whereas the low-density organic/inorganic coating and the metallic scaffold present a challenging contrast imbalance. In addition, beam-hardening and edge artifacts from the metal substrate may obscure the coating/substrate interface and lead to large uncertainty in local thickness extraction. Therefore, micro-CT was considered unsuitable for reliable quantitative evaluation of coating thickness and variability in the present system. Instead, coating homogeneity was evaluated by replicate coating-mass measurements, calculated reference thickness, and optical images. The relative mass errors among three replicate samples were 1.06%, 1.86%, and 1.16% for Gyroid, Primitive, and Diamond, respectively, and the coating-mass deviation between TPMS contactors and the Square contactor was below 3%. Given that all substrates were designed with identical specific surface area, these results confirm that the TPMS and Square contactors had comparable adsorbent loadings.

**Supplementary Note 1**

CO_2_ breakthrough experiments were conducted on the contactors using a custom-built fixed-bed reactor with a diameter of 18 mm (Supplementary Fig. 5). All substrate profiles are cylindrical with a diameter of 18 mm and a height of 30 mm (Supplementary Fig. 6 Supplementary Fig. 7). The coating quality of contactors with different structures was shown in the Supplementary Table 2. During the 3-hour adsorption process, a 400 ppm CO_2_/N_2_ gas mixture was introduced at flow rates of 1 and 2 L min^−1^. The CO_2_ concentration in the outlet gas was monitored using an infrared gas analyzer. The dynamic breakthrough curves in Supplementary Fig. 9 shows a strong positive correlation between the total adsorption time of a contactor and its adsorbent loading. The breakthrough point, half-saturation point, and saturation point are defined as the points on the breakthrough curve where the outlet CO_2_ concentration reaches 5%, 50%, and 95% of the inlet concentration, respectively. The time required to reach these points is termed the breakthrough time (*t*_5_), half-saturation time (*t*_50_), and the saturation time (*t*_95_).

To achieve adsorption, the adsorbed molecules need to go through the following processes: first, they need to reach the adsorbent particles through convection; then, they diffuse in the fluid film surrounding the particles; subsequently, they diffuse longitudinally along the pores until they find vacant adsorption sites; finally, they are adsorbed on the solid surface. Assuming that the adsorption bed is in a clean state at the beginning of the operation, meaning that the adsorbent has been fully regenerated and does not contain any adsorbate. When the feed gas is introduced, the mass transfer process immediately commences, causing the concentration of the adsorbate to gradually decrease along the length of the bed until it approaches zero concentration. Meanwhile, fresh feed gas continuously enter the reactor, ensuring that the initial part of the bed layer that comes into contact with the feed gas remains in a fluid environment with the feed gas concentration. Eventually, this part of the bed layer will reach an equilibrium state with the feed gas, and no additional net mass transfer will occur at this point. When the feed gas flow reaches the bed layer area that has not yet reached equilibrium, the mass transfer process restarts. As the bed layer length increases, the fluid concentration decreases again, approaching zero.

This region of concentration change is called the mass transfer zone (MTZ). As the reaction process progresses, the contact between the adsorbent and the feed gas gradually approaches equilibrium. The feed gas needs to continuously advance to reach the bed layer area that has not yet reached equilibrium. Through this dynamic change, the mass transfer zone continuously extends along the length of the bed layer, and the proportion of the utilized area in the bed layer also continuously increases. Before the mass transfer breakthrough, the concentration of the adsorbed substance in the effluent is almost zero. As the mass transfer zone gradually moves away from the top of the tower, the concentration begins to gradually increase. When the rear edge of the mass transfer front reaches the end of the tower bed, the concentration of the effluent eventually reaches the feed concentration. This process occurs during the equilibrium time, at which point the entire tower bed is in complete equilibrium with the feed. Therefore, the length of the MTZ (the difference between *t*_5_ and *t*_95_) depends on the mass transfer rate.

In an ideal situation, if there is no resistance to mass transfer, the mass transfer will be completed instantaneously, and at this point, the length of the mass transfer zone approaches zero. This zone will advance in a flat-front form, and the bed layer will have reached a completely balanced state during the breakthrough, and the breakthrough curve will be a straight line. This trend indicates that an increase in coating thickness results in a slower propagation of the adsorption front and a broader MTZ.

Supplementary Table 3. Comparison of *t*_5_, *t*_50_, and *t*_95_ for Square and TPMS contactors at 1 and 2 L/min

| Contactor | *t*_5_  (min) | *t*_50_  (min) | *t*_95_  (min) | MTZ (*t*_95_ − *t*_5_)  (min) |
| --- | --- | --- | --- | --- |
| Square− | 2.6/1.1 | 6.7/4.2 | 45.7/14.9 | 43.1/13.8 |
| Square | 17.6/2.2 | 32.2/10.7 | 133.7/86.9 | 116.1/84.7 |
| Square+ | 20.4/2.6 | 45.4/14.2 | 300.5/168.1 | 280.1/165.5 |
| Gyroid | 26.2/15.7 | 39.9/25.1 | 152.2/65.3 | 126.0/49.6 |
| Primitive | 17.6/8.8 | 41.6/19.3 | 129/99.2 | 111.4/90.4 |
| Diamond | 27.1/13.7 | 36.5/21.4 | 130.3/61.5 | 103.2/47.8 |

The number before the “/” represents a flow rate of 1 L/min, while the number following the “/” indicates a flow rate of 2 L/min.

Supplementary Fig. 10 (a) Volumetric capacity as a function of time. (b) Volumetric capacity as a function of the normalized outlet concentration. (c) Gravimetric capacity as a function of the normalized outlet concentration.

Supplementary Table 4. Total gravimetric adsorption capacities of contactors

| Contactor | Adsorbent mass (Coating composite) (g) | CO_2_ uptake (mmol) | Gravimetric capacity  (mmol/g) |
| --- | --- | --- | --- |
| Square− | 0.184 | 0.25/0.22 | 1.36/1.20 |
| Square | 0.692 | 0.84/0.78 | 1.21/1.13 |
| Square+ | 1.161 | 1.27/1.18 | 1.09/1.02 |
| Gyroid | 0.706 | 1.00/1.18 | 1.42/1.67 |
| Primitive | 0.680 | 0.96/1.13 | 1.41/1.66 |
| Diamond | 0.695 | 0.91/1.05 | 1.31/1.51 |

**Note:** Adsorbent mass refers to the total mass of the coated composite layer (SBS-PEI/MMO), excluding the metallic substrate; gravimetric capacities are normalized per gram of coating (mmol gcoating^−1^).

The number before the “/” represents a flow rate of 1 L/min, while the number following the “/” indicates a flow rate of 2 L/min.

**Supplementary Note 2**

Supplementary Fig. 10a presents the volumetric adsorption capacity as a function of time for each contactor. A striking difference exists between the TPMS-based contactors and the straight-channel Square contactors. The Gyroid, Primitive, and Diamond structures exhibit vastly superior adsorption rates, reaching near-saturation capacity of 140 mol m^-3^ within 100 minutes. The rapid kinetics of TPMS structures are fundamental to achieving high productivity. In contrast, even the best-performing straight-channel Square+ contactor requires over 180 minutes to approach a similar capacity, while the Square and Square- variants show significantly slower uptake. Within the Square family, an inverse relationship between adsorbent loading and kinetic rate is observed.

Supplementary Fig. 10b displays volumetric capacity against the normalized outlet CO_2_ con-centration. The steepness of the breakthrough curve is a direct indicator of the MTZ length. The Square contactors, especially Square- and Square, exhibit a very broad and shallow S-shaped curve. For these contactors, a substantial portion of their total volumetric capacity is attained only at high outlet concentration, meaning the CO_2_ capture rate has dropped. This represents an unfavorable trade-off that leads to poor utilization of the contactor’s potential capacity. The TPMS structures display step-like breakthrough curves, demonstrating their ability to maintain a high capture rate until achieving a high volumetric capacity. This characteristic is desirable as it allows the process to be operated efficiently for the majority of the adsorption step, maximizing both the quantity of CO_2_ captured and the capture rate.Supplementary Fig. 10c shows the gravimetric capacity against the normalized outlet concentration. For square-channel contactors, accessing high fractions of the intrinsic capacity over 1.2 mmol g^−1^ necessitates tolerating high outlet concentration, indicating inefficient utilization of the active material during practical, high-efficiency operation. Conversely, the steep curves of the TPMS structures indicate that a high fraction of the intrinsic capacity can be effectively utilized while maintaining a considerable capture rate. This demonstrates that the enhanced mass transfer within TPMS geometries improves overall contactor performance and maximizes the working efficiency of the adsorbent material.

# Section 3 Modeling adsorption

Supplementary Table 5. Fitted Langmuir Isotherm Parameters

| T（K） |  |  |
| --- | --- | --- |
| 298.15 | 1.962 | 332.95266 |
| 318.15 | 2.69594 | 80.23438 |
| 338.15 | 3.4546 | 7.82265 |

Supplementary Fig. 11 Arrhenius-type Temperature Dependence of Langmuir Constant $\boldsymbol{k}_{\mathbf{L}}\boldsymbol{=1.4\times}\boldsymbol{10}^{\boldsymbol{-8}}\mathbf{exp}\left( \mathbf{-}\frac{\boldsymbol{59235}}{\boldsymbol{RT}} \right)$

Supplementary Table 6. Governing equations for fixed-bed adsorption model

| **Feature** | **General Equation** |
| --- | --- |
| Mass Balance | $\frac{\partial\left( uC_{i} \right)}{\partial z}+\frac{\partial C_{i}}{\partial t}+N_{i}=D_{L}\frac{\partial}{\partial z}\left( \frac{\partial C_{i}}{\partial z} \right) \forall z\in\left( 0,L \right),i=1,\ldots,N_{\text{comp }} (S2)$  $N_{\mathrm{CO}_{2}}=\frac{{As}_{1}}{{As}_{2}}\rho_{\mathrm{ads}}\frac{d(q_{1}+q_{2})}{dt} (S3)$ |
| Momentum Balance | $-\frac{\partial P}{\partial z}=\frac{8\mu u}{R_{1}^{2}} \forall z\in\left( 0,L \right) (S4)$ |
| Heat Balance | $\begin{matrix} \frac{\partial\left( \epsilon_{\mathrm{bed}}\rho c_{p}uT \right)}{\partial z}+\frac{\partial\left( \epsilon_{\mathrm{bed}}\rho c_{p}T \right)}{\partial t}+\left( 1-\epsilon_{\mathrm{bed}} \right)q_{g}+\frac{3k_{h,\mathrm{wall}}}{R_{\mathrm{bed}}}\left( T-T_{\mathrm{wall}} \right)= & \\ \epsilon_{\mathrm{bed}}\frac{\partial}{\partial z}\left( \lambda_{z}\frac{\partial T}{\partial z} \right), \forall z\in\left( 0,L \right) & \end{matrix}$ $(S5)$ |

**Supplementary Note 3**

The boundary conditions applied to solve the model are summarized in Supplementary Table 7. At the inlet (*z* = 0), feed conditions govern gas composition and flow properties. At the outlet (*z* = *L*), the pressure is maintained at atmospheric conditions, while thermal and concentration gradients follow convective flux principles.

Supplementary Table 7. Boundary Condition

| Inlet *z* = 0 | Outlet *z* = *L* |
| --- | --- |
| $D_{L}\frac{\partial C_{i}\left( 0 \right)}{\partial z}=-u_{\mathrm{feed}}C_{\mathrm{feed},i}+u\left( 0 \right)C_{i}\left( 0 \right)$  $u\left( 0 \right)C\left( 0 \right)$=$u_{\mathrm{feed}}C_{\mathrm{feed}}$ | $\frac{\partial C_{i}(L)}{\partial z}=0$  $\frac{\partial u(L)}{\partial z}=0$ |
| $\frac{\partial p\left( 0 \right)}{\partial z}=-\frac{8\mu_{\mathrm{air}}v\left( 0 \right)}{R_{\mathrm{bed}}^{2}}$ | $p(L)=p_{\mathrm{atm}}$ |
| $k_{\mathrm{air}}\frac{\partial T_{\mathrm{gas}}\left( 0 \right)}{\partial z}=\rho_{\mathrm{air}}C_{p,\mathrm{air}}u\left( 0 \right)T_{\mathrm{gas}}\left( 0 \right)-\rho_{\mathrm{air}}C_{p,\mathrm{air}}u_{\mathrm{feed}}T_{\mathrm{feed}}$ | $\frac{\partial T_{\mathrm{gas}}(L)}{\partial z}=0$ |

Supplementary Table 8. Key Model Symbols

| Symbol | Description | Unit |  |
| --- | --- | --- | --- |
| $\boldsymbol{q}_{\boldsymbol{e}}$ | Equilibrium Adsorption Capacity | mol/kg | 0 –1.962 |
| $\boldsymbol{q}_{\boldsymbol{m}}$ | Maximum Adsorption Capacity (Langmuir) | mol/kg | 1.962 |
| $\boldsymbol{q}_{\boldsymbol{1}}\boldsymbol{、}\boldsymbol{q}_{\boldsymbol{2}}$ | Instantaneous Adsorption Capacity | mol/kg | 0 – *q_e_* |
| $\boldsymbol{k}_{\boldsymbol{L}}$ | Langmuir Equilibrium Constant | Pa^−1^ | Computed from Eq. (2) |
| $\boldsymbol{P}\boldsymbol{、}\boldsymbol{P}_{\boldsymbol{CO}_{\boldsymbol{2}}}$ | Total Pressure, CO₂ Partial Pressure | Pa | *P* ≈ 1.013×10^5^;  *P*_CO2_: 0 – 40.5 |
| $\boldsymbol{T}$ | Temperature | K | 298.15 – 373.15 |
| $\boldsymbol{k}_{\boldsymbol{0}}$ | Arrhenius Pre-exponential Factor (Langmuir) | Pa^−1^ | 1.4×10^−8^ |
| $\boldsymbol{E}$ | Enthalpy of Adsorption | J/mol | −59200 |
| $\boldsymbol{R}$ | Gas Constant | J/mol/K | 8.314 |
| $\boldsymbol{k}\boldsymbol{k}_{\boldsymbol{1}} \boldsymbol{k}_{\boldsymbol{2}}$ | LDF Mass Transfer Coefficient |  | Fitted per contactor; see Fig. 3f–3h, Suppl. Table 10 |
| $\boldsymbol{\eta}$ | Fraction of Capacity in Fast Kinetic Domain |  | Fitted per contactor; see Fig. 3f–3h, Suppl. Table 10 |
| $\boldsymbol{t}$ | Time | s | 0 – t_95_ |
| $\boldsymbol{C}_{\boldsymbol{i}}$ | Molar Concentration of Component i | mol/m^3^ | 0 – 0.01635 |
| $\boldsymbol{u}$ | Superficial Gas Velocity | m/s | Model-resolved |
| $\boldsymbol{u}_{\boldsymbol{feed}}$ | Feed Gas Velocity | m/s | 0.066–0.131 |
| $\boldsymbol{C}_{\boldsymbol{feed}}$ | Feed Gas Molar Concentration | mol/m^3^ | 0.01635 (CO_2_) |
| $\boldsymbol{T}_{\mathbf{feed}}$ | Feed Gas Temperature | K | 298.15 |
| $\boldsymbol{N}_{\boldsymbol{i}}$ | Adsorption Rate (Source/Sink Term) | mol/m^3^/s | Model-resolved |
| $D_{L}$ | Axial Dispersion Coefficient | m^2^/s | 1×10^−5^ |
| $\boldsymbol{L}$ | Bed Length | m | 0.03 |
| $\boldsymbol{\mu}$ | Gas Dynamic Viscosity | Pa·s | 1.81×10^−5^ |
| $\boldsymbol{As}_{\boldsymbol{1}}\boldsymbol{、}\boldsymbol{As}_{\boldsymbol{2}}$ | Equivalent Hydraulic Radius | m | see Table 1 |
| $\boldsymbol{R}_{\boldsymbol{bed}}$ | Bed Radius | m | 0.009 |
| $\boldsymbol{\rho}$ | Gas Density | kg/m^3^ | 0.955~1.178 |
| $\boldsymbol{c}_{\boldsymbol{p}}$ | Gas Specific Heat Capacity (const. P) | J/kg/K | 1005 |
| $\boldsymbol{q}_{\boldsymbol{g}}$ | Volumetric Adsorption Heat Source | W/m^3^ | Model-resolved |
| $\boldsymbol{k}_{\mathbf{h}\boldsymbol{,}\mathbf{wall}}$ | Wall Heat Transfer Coefficient | W/m^2^/K | 15 |
| $\boldsymbol{k}_{\mathbf{wall}}$ | Wall Temperature | K | Adsorption 298.15 Desorption 373.15 |
| $\boldsymbol{\lambda}_{\boldsymbol{z}}$ | Effective Axial Thermal Conductivity | W/m/K | 0.3 |

Note: For dependent (model-resolved) variables, the Value column lists the bounds spanned during the simulation. These quantities are uniquely determined by the tabulated inputs and the governing equations (Eqs. 1–5) and are not adjustable parameters. The dual-LDF parameters *k*_1_, *k*_2_ and the fast-site fraction η are obtained by fitting the experimental breakthrough curve of each contactor (Fig. 3f–3h, Supplementary Table 10).

Supplementary Fig. 12 Fitting results of breakthrough curves for square contactors.

Supplementary Fig. 13 Fitting results of breakthrough curves for TPMS contactors.

Supplementary Table 9. R_2_ values of contactor breakthrough curve fitting results

|  | 1 L min^−1^ | 2 L min^−1^ |
| --- | --- | --- |
| Square− | 0.994 | 0.9957 |
| Square | 0.9978 | 0.9836 |
| Square+ | 0.999 | 0.9952 |
| Gyroid | 0.9979 | 0.9870 |
| Primitive | 0.9989 | 0.9945 |
| Diamond | 0.9995 | 0.9990 |

Supplementary Table 10. Relevant mass transfer parameter values fitted by adsorption model

|  | 1 L min^−1^ | | | 2 L min^−1^ | | |
| --- | --- | --- | --- | --- | --- | --- |
|  | *η* | *k*_1_ | *k*_2_ | *η* | *k*_1_ | *k*_2_ |
| Square− | 0.65 | 6.8 | 0.80 | 0.64 | 19 | 1.5 |
| Square | 0.57 | 4.1 | 0.33 | 0.44 | 5.6 | 0.35 |
| Square+ | 0.36 | 2.8 | 0.27 | 0.31 | 3.9 | 0.21 |
| Gyroid | 0.59 | 5.1 | 0.77 | 0.77 | 7.5 | 2.7 |
| Primitive | 0.71 | 2.4 | 0.61 | 0.68 | 4.9 | 0.84 |
| Diamond | 0.63 | 5.4 | 0.48 | 0.75 | 7.0 | 1.5 |

# Section 4 Pressure drop

**Geometric Model Establishment**

**Straight-channel geometric model:** The straight-channel model was constructed entirely using the built-in “Geometry Creation Tool” in the COMSOL Multiphysics software. As the straight-channel model is geometrically symmetric, only a single channel was modeled to save computational resources and reduce calculation time.

**TPMS structure geometric model:** The model was generated using the open-source MATLAB plugin MSLattice, with geometric parameters consistent with those described in Table 1. Because the generated model is in STL format, which results in slow geometric import into COMSOL Multiphysics, and since TPMS models are periodic structures, only single unit cells of the different TPMS structures were generated in MSLattice and then duplicated along the axial direction using the “Array” tool in COMSOL Multiphysics. Due to the complexity of the TPMS structures and the numerous boundary conditions, CFD calculations were performed only on contactor models with 5, 10, 15, and 20 axial unit cells. The pressure drop values obtained from these simulations were then fitted against length to calculate the pressure drop for the target contactor length.

**Boundary Condition Settings:** The material for the fluid domain was set to “Air” from the COMSOL Multiphysics built-in material library, with all fluid properties derived from the material’s physical parameters. The airflow inlet condition was set to a normal inflow velocity of *vel* m s^−1^, the airflow outlet was set to a pressure boundary condition of 0 Pa with backflow suppressed, and the global pressure was set to 101325 Pa.

**Contactor Walls:** A no-slip boundary condition was applied to the walls of both the Square and TPMS contactors. For the TPMS models, periodic boundary conditions were set on the laterally open boundaries of the flow channels.

**Computational Settings:**The laminar flow model was used for solving. All models were computed under steady-state conditions.

**Meshing and Grid Independence Verification**

A physics-controlled mesh was generated using COMSOL Multiphysics. Boundary layer meshes were generated on the contactor walls, and a free tetrahedral mesh was created for the remaining fluid domain. Taking the 5-unit-cell Gyroid structure model as an example, the number of generated mesh elements was 441,986, with an average mesh quality of 0.6758 (measured by skewness), which satisfied the computational requirements. A grid independence verification was conducted using the 5-unit-cell Gyroid model with an inlet air velocity of 1 m s^−1^ as the baseline. The pressure difference between the inlet and outlet was calculated for meshes with 1.2×10^5^, 2.2×10^5^, 4.4×10^5^, and 11.9×10^5^ elements. The pressure drop value from the 11.9×10^5^ element mesh was used as the benchmark, and the results are shown in Supplementary Table 11. When the mesh count was 4.4×10^5^, the calculated error in pressure drop was 3.72%, which is within an acceptable margin. Therefore, to ensure computational accuracy while conserving resources and reducing complexity, this mesh configuration was adopted as the standard for all subsequent simulations. In the subsequent calculations, the meshing settings for all models were identical to those used to generate the 4.4×10^5^ element mesh for the 5-unit-cell Gyroid structure model.

Supplementary Fig. 14 Grid display (Gyroid)

Supplementary Table 11. Pressure drop in the five-cell model of Gyroid with different grid numbers

| Number of Elements | 115067 | 218725 | 441986 | 1189483 |
| --- | --- | --- | --- | --- |
| Pressure Drop (Pa) | 22.91 | 21.17 | 20.06 | 19.34 |
| Error | 18.5% | 9.46% | 3.72% | 0% |

**Experimental Validation**

Airflow was generated by a variable-voltage fan, and its velocity was measured after passing through a flow equalizer. The air was then channeled into the gas-solid contactor model to be tested. The pressure drop was measured using a differential pressure transmitter with probes placed 2 cm upstream of the inlet and 2 cm downstream of the outlet. The equipment used included a JYF-50S fan (6W, 330 Pa air pressure; 18W, 570 Pa air pressure), a PCM620Y differential pressure transmitter (range: −800 to 800 Pa, accuracy: 0.5% FS), and an SW6086 thermal anemometer (range: 0.0 to 30.0 m s^−1^, accuracy: 10% FS).

The contactor models were fabricated using a Bambu Lab P1S 3D printer with PLA material. However, due to the limited precision of the printer, the geometric dimensions specified in Table 1 were uniformly scaled up by a factor of three to ensure the model could be accurately formed.

The specifications of the fabricated model were 300 mm in length, 50 mm in width, and 50 mm in height, which was 3 times scale up compare to original prototype model. The principle of similarity was used to perform an equivalent calculation for the pressure drop between the experimental model and the original prototype. As the process only involves gas flow, the equivalent calculation correlating the experimentally measured pressure drop of the scaled-up model to that of the actual prototype was based on the principle of Reynolds number similarity and its corresponding formula.

Supplementary Fig. 15 Pressure drop experimental setup

Supplementary Fig. 16 Comparative analysis of gas flow vorticity distributions within the Square and TPMS contactors based on CFD simulation data.

Supplementary Fig. 17 Experimental simulation verification comparison

As shown in Supplementary Fig. 17, the converted experimental pressure drops agree well with the CFD predictions for the Square and TPMS contactors over the tested velocity range. This comparison confirms that the CFD model captures the relative pressure-drop trends among the different contactor geometries and supports its use in the fan-energy and performance evaluation.

**Pressure drop estimation for a packed-bed reference**

The pressure drop of a packed-bed reference contactor was estimated using the Ergun equation, which accounts for both viscous and inertial contributions to gas flow through a randomly packed particle bed:

$$\frac{\Delta P}{L}=\frac{150\mu(1-\varepsilon)^{2}}{\varepsilon^{3}d_{p}^{2}}u_{s}+\frac{1.75\rho(1-\varepsilon)}{\varepsilon^{3}d_{p}}u_{s}^{2}$$

where $\Delta P$is the pressure drop, $L$is the bed length, $\mu$is the dynamic viscosity of air, $\rho$is the density of air, $\varepsilon$is the bed void fraction, $d_{p}$is the particle diameter, and $u_{s}$is the superficial gas velocity based on the empty column cross-sectional area.

The parameters used in the calculation are listed below:

Supplementary Table 12. Parameters for pressure drop calculation

| **Parameter** | **Value** | **Unit** |
| --- | --- | --- |
| Bed length, $L$ | 0.30 | m |
| Particle diameter, $d_{p}$ | $3.0\times{10}^{-3}$ | m |
| Bed void fraction, $\varepsilon$ | 0.40 | — |
| Air dynamic viscosity, $\mu$ | $1.85\times{10}^{-5}$ | Pa s |
| Air density, $\rho$ | 1.184 | Kg/m^3^ |

Substituting these values into the Ergun equation gives:

$$\frac{\Delta P}{L}=\frac{150(1.85\times{10}^{-5})(1-0.40)^{2}}{{0.40}^{3}(3.0\times{10}^{-3})^{2}}u_{s}+\frac{1.75(1.184)(1-0.40)}{{0.40}^{3}(3.0\times{10}^{-3})}u_{s}^{2}$$

Therefore,

$$\frac{\Delta P}{L}=1734.4u_{s}+6475.0u_{s}^{2}$$

where $\Delta P/L$is in Pa/m and $u_{s}$is in m/s.

The particle Reynolds number can be estimated as:

$$Re_{p}=\frac{\rho u_{s}d_{p}}{\mu}$$

Under the velocity range considered in this work, both viscous and inertial terms were retained in the pressure-drop calculation.

Supplementary Table 13. Fan-energy contribution and fan-energy-normalized CO_2_ capture for different contactor structures.

|  | **E_fan_ (GJ/t_CO2_)** | **CO_2_ captured per fan energy**  **(t_CO2_/GJ_fan_)** |
| --- | --- | --- |
| Square− | 0.100 | 10.00 |
| Square | 0.104 | 9.62 |
| Square+ | 0.107 | 9.35 |
| Gyroid | 1.446 | 0.69 |
| Diamond | 0.872 | 1.15 |
| Primitive | 2.218 | 0.45 |

# Section 5 Performance evaluation

Supplementary Table 14. Modeling parameters of scale-up contactor of performance evaluation

| Parameters | Value | Unit |
| --- | --- | --- |
| Velocity | 1.3 | m·s^−1^ |
| Length of contactor | 300 | mm |
| Volume | 1 | m^3^ |
| Substrate density | 2680 | kg·m^−3^ |
| Specific heat capacity of substrate | 0.75 | kJ·kg^−1^·K^−1^ |
| Specific heat capacity of adsorbent | 1.16 | kJ·kg^−1^·K^−1^ |
| Specific heat capacity of N_2_ | 1.04 | kJ·kg^−1^·K^−1^ |
| Heat of adsorption of adsorbent | 57.28 | kJ·mol^−1^ |
| Adsorption temperature | 25 | °C |
| Regeneration temperature | 120 | °C |

**Supplementary Note 4**

Decoupling heat and mass transfer during desorption. The characteristic conduction time is t ≈ L^2^ρcp/k. For AlSi10Mg (k ≈ 150 W m^−1^ K^−1^, ρ ≈ 2670 kg m^−3^, cp ≈ 900 J kg^−1^ K^−1^), this gives ≈0.4 ms across the 150 μm wall and ≈1–2 s across the 9 mm contactor radius, both far shorter than the tens-of-minutes desorption time. The N_2_ purge (100 mL/min) is preheated to the 120 °C regeneration temperature; even under the bounding assumption that it entered at 25 °C, its heat input would be ṁcpΔT ≈ (1.9×10^−6^ kg s^−1^)(1.04 kJ kg^−1^ K^−1^)(95 K) ≈0.2 W, which is negligible. The purge therefore serves a mass-transfer role (sweeping desorbed CO_2_ and lowering the CO_2_ partial pressure at the sorbent surface), not a heat-delivery role.

**Supplementary Note 5**

The performance of the contactor was evaluated by calculating key performance indicators during the adsorption process, primarily including the specific productivity (*P*, mol_CO2_ m^−3^ d^−1^) and specific energy consumption (*W*_total_, GJ t _CO2_^−1^, which consists of the sensible heat for the adsorbent and contactor (E_sensible_heat_), heat required for the nitrogen purge gas (E_purge_), the heat of desorption for CO_2_ (E_adsorption_heat_), and fan energy (E_fan_).

The calculation formula is as follows:

$$\begin{aligned} E_{\text{sensible}\text{\_}\text{heat}}=\frac{\left( m_{\text{ads}}\cdot C_{p,\text{ads}}+m_{\text{sub}}\cdot C_{p,\text{sub}} \right)\cdot\left( T_{\text{des}}-T_{\text{ads}} \right)}{m_{\text{CO}_{2},\text{cyc}}} \# \end{aligned}(S6)$$

$$\begin{aligned} E_{\text{purge}}=\frac{m_{\text{purge}}\cdot C_{p,\text{purge}}\cdot\left( T_{\text{des}}-T_{\text{ads}} \right)}{m_{\text{CO}_{2},\text{cyc}}} (S7)\# \end{aligned}$$

$$\begin{aligned} E_{\mathrm{fan}}=\frac{\Delta P\cdot V_{\text{air}}}{m_{\text{CO}_{2},\text{cyc}}\cdot\eta_{\text{fan}}} \# \end{aligned}(S8)$$

$$\begin{aligned} P=\frac{q_{\mathrm{ads}}}{{(t}_{\mathrm{ads}}+t_{\mathrm{de}s})\cdot V\mathrm{cont}\mathrm{actor}} (S9) \# \end{aligned}$$

$\begin{aligned} q_{\mathrm{ads}}=\frac{\int_{0}^{t_{\mathrm{ads}}} \left( \dot{n}_{\mathrm{in}}-\dot{n}_{\mathrm{out}} \right)dt-n_{\mathrm{col}}}{m_{ads}}\# \end{aligned}$(S10)

$\begin{aligned} n_{\mathrm{col}}=\frac{y_{\mathrm{in}}p_{\mathrm{col}}\varepsilon^{*}V_{\mathrm{col}}}{RT_{\mathrm{col}}}\# \end{aligned} (S11)$

$$\begin{aligned} r=\frac{q_{\mathrm{ads}}m_{\mathrm{ad}s}}{\int_{0}^{t_{\mathrm{ads}}} \dot{n}_{\mathrm{in}}dt}\# \end{aligned} (S12)$$

Where:

$m_{\text{ads}}$: Total mass of the adsorbent in the contactor (kg).

$C_{p,\text{ads}}$: Specific heat capacity of the adsorbent material (J·kg^−1^·K^−1^).

$m_{\text{sub}}$: Total mass of the contactor substrate (kg).

$C_{p,\text{sub}}$: Specific heat capacity of the substrate material (J·kg^−1^·K^−1^).

$T_{\text{des}}$: Desorption (regeneration) temperature (K).

$T_{\text{ads}}$: Adsorption temperature (K).

$m_{\text{CO}_{2},\text{cyc}}$: Total mass of CO_2_ captured per adsorption-desorption cycle (t).

$m_{\text{purge}}$: Total mass of the purge gas (e.g., N_2_) used per cycle (kg).

$C_{p,\text{purge}}$: Specific heat capacity of the purge gas (J·kg^−1^·K^−1^).

ΔP: The total pressure drop of the air as it flows across the contactor (Pa). Determined by a CFD analysis.

$V_{\text{air}}$: The total volume of air processed during the adsorption step of a single cycle (m^3^).

$\eta_{\text{fan}}$: The overall efficiency of the fan and motor system (0.5).

$q_{\mathrm{ads}}$: The adsorption capacity at *t*_ads_ (mmol g^−1^).

$\dot{n}$and$p$: The molar flow rate of CO_2_ and the pressure measured at the inlet and outlet of the fixed bed.

Supplementary Table 15. Normalized quantitative KPI of contactor performance evaluation.

|  | Square− | Square | Square+ | Gyroid | Primitive | Diamond |
| --- | --- | --- | --- | --- | --- | --- |
| Adsorption capacity per unit volume | 0.153 | 0.380 | 0.486 | 1.000 | 0.721 | 0.855 |
| Adsorption kinetics | 0.922 | 0.888 | 0.861 | 0.996 | 0.931 | 1.000 |
| Adsorbent utilization rate (Working capacity of coating adsorbent) | 0.587 | 0.387 | 0.295 | 1.000 | 0.748 | 0.869 |
| Desorption kinetics | 0.139 | 0.238 | 0.261 | 1.000 | 0.579 | 0.852 |
| Productivity | 0.329 | 0.607 | 0.695 | 1.000 | 0.861 | 0.956 |
| Energy efficiency  =1/Energy consumption | 0.216 | 0.426 | 0.455 | 1.000 | 0.790 | 0.911 |

*Note:* For beneficial metrics, normalized values were calculated as x/x_max_. For penalty metrics, including desorption time and specific energy consumption, inverse metrics were used before normalization, e.g., x_min_/x. Thus, a larger normalized value consistently indicates better performance in the radar chart.

Supplementary Table 16. Unnormalized raw values for KPIs of contactor performance evaluation.

|  | Square- | Square | Square+ | Gyroid | Primitive | Diamond |
| --- | --- | --- | --- | --- | --- | --- |
| Adsorption capacity per unit volume | 16.543 | 41.184 | 52.676 | 108.396 | 78.122 | 92.706 |
| Adsorption kinetics | 4.133 | 10.683 | 14.083 | 25.067 | 19.333 | 21.350 |
| Working capacity of coating adsorbent | 0.688 | 0.454 | 0.346 | 1.172 | 0.877 | 1.019 |
| Desorption kinetics | 26.917 | 39.167 | 45.667 | 24.500 | 30.500 | 24.583 |
| Productivity | 48.0 | 88.5 | 101.3 | 145.8 | 125.5 | 139.4 |
| Energy consumption | 48.3 | 24.7 | 22.9 | 11.9 | 13.226 | 12.34 |

Supplementary Table 17. Volumetric and mass-normalized CO_2_ productivity of all contactors (t_ads_ = t_50_, t_des_ = t_80_).

| Contactor | Volumetric productivity  (kg /m^3^/d) | Mass-normalized productivity per coating adsorbent  (kg_CO2_/kg_coating_adsorbent_/d) | Mass-normalized productivity per contactor  (kg_CO2_/kg_total_contactor_/d) |
| --- | --- | --- | --- |
| Square− | 48.0 | 1.990 | 0.178 |
| Square | 88.5 | 0.976 | 0.263 |
| Square+ | 101.3 | 0.666 | 0.255 |
| Gyroid | 145.8 | 1.580 | 0.408 |
| Primitive | 125.5 | 1.410 | 0.372 |
| Diamond | 139.4 | 1.530 | 0.409 |

Supplementary Table 18. Model-based comparison of a fiber-type contactor and TPMS contactors.

| **Active sorbent fraction** | **Axial flow (no lateral mixing)** | **TPMS lateral mixing** |
| --- | --- | --- |
| Metallic substrate (current) | 24.7 | 11.9 |
| Zero inactive mass (fiber-like limit) | 14.4 | 8.0 |

**Note:** Model-based comparison of a fiber-type contactor and TPMS contactors, with active sorbent fraction and flow topology varied independently. Values are total specific energy (GJ/t_CO2_) under identical dry-N_2_, no-heat-recovery conditions and matched active sorbent loading. The fiber-type contactor is represented by the square-channel kinetics and pressure drop (axial flow without TPMS secondary-flow mixing); the zero-inactive-mass row is the limiting case of the Fig. 5d wall-thickness analysis.

Because the wall-thickness sensitivity analysis (Fig. 5d) was computed with the adsorption capacity, fitted kinetic parameters, pressure drop, and cycle conditions held fixed, the total specific energy is linear in the substrate wall thickness, with slopes of 0.0688 and 0.0262 GJ/t_CO2_/μm for the Square and Gyroid geometries, respectively. At the 150 μm baseline, the substrate sensible-heat contribution is therefore ≈10.3 GJ/t_CO2_ for the Square geometry (0.0688 × 150) and ≈3.9 GJ/t_CO2_ for the Gyroid geometry (0.0262 × 150).

The zero-inactive-mass (fiber-like) limits reported in Supplementary Table 18 are obtained by extrapolating each linear fit to zero substrate thickness, which removes this substrate sensible-heat share while retaining the corresponding fan-energy term. For the axial-flow, square-channel geometry used to represent a fiber-type contactor, the 150 μm baseline of 24.7 GJ/t_CO2_ is reduced to ≈14.4 GJ/t_CO2_; for the Gyroid (porous-wall TPMS) geometry, the baseline of 11.9 GJ/t_CO2_ is reduced to 8.0 GJ/t_CO2_.

Thus, even in the idealized zero-inactive-mass limit, the fiber-type (axial-flow) configuration (≈14.4 GJ/t_CO2_) remains more energy-intensive than the porous-wall TPMS configuration (8.0 GJ/t_CO2_). Moreover, the current metal-substrate Gyroid contactor (11.9 GJ/t_CO2_) already consumes less energy per ton of CO_2_ than the fiber-type limit, despite carrying its full inactive substrate mass and fan-energy penalty. This isolates the lateral-mixing contribution of the TPMS topology from the active-sorbent-fraction (inactive-mass) contribution exploited by fiber sorbents.

# Reference

1.	Zhu, X. *et al.* Efficient CO2 capture from ambient air with amine-functionalized Mg–Al mixed metal oxides. *J. Mater. Chem. A* **8**, 16421–16428 (2020).
